# Protein kinase Cε regulates nuclear translocation of extracellular signal-regulated kinase, which contributes to bradykinin-induced cyclooxygenase-2 expression

**DOI:** 10.1038/s41598-018-26473-7

**Published:** 2018-06-04

**Authors:** Rei Nakano, Taku Kitanaka, Shinichi Namba, Nanako Kitanaka, Hiroshi Sugiya

**Affiliations:** Laboratory of Veterinary Biochemistry, Department of Veterinary Medicine, Nihon University College of Bioresource Sciences, 1866 Kameino, Fujisawa, Kanagawa 252-0880 Japan

## Abstract

The proinflammatory mediator bradykinin stimulated cyclooxygenase-2 (COX-2) expression and subsequently prostaglandin E_2_ synthesis in dermal fibroblasts. The involvement of B2 receptors and Gαq in the role of bradykinin was suggested by using pharmacological inhibitors. The PKC activator PMA stimulated COX-2 mRNA expression. Bradykinin failed to induce COX-2 mRNA expression in the presence of PKC inhibitors, whereas the effect of bradykinin was observed in the absence of extracellular Ca^2+^. Bradykinin-induced COX-2 mRNA expression was inhibited in cells transfected with PKCε siRNA. These observations suggest that the novel PKCε is concerned with bradykinin-induced COX-2 expression. Bradykinin-induced PKCε phosphorylation and COX-2 mRNA expression were inhibited by an inhibitor of 3-phosphoinositide-dependent protein kinase-1 (PDK-1), and bradykinin-induced PDK-1 phosphorylation was inhibited by phospholipase D (PLD) inhibitors, suggesting that PLD/PDK-1 pathway contributes to bradykinin-induced PKCε activation. Pharmacological and knockdown studies suggest that the extracellular signal-regulated kinase 1 (ERK1) MAPK signaling is involved in bradykinin-induced COX-2 expression. Bradykinin-induced ERK phosphorylation was attenuated in the cells pretreated with PKC inhibitors or transfected with PKCε siRNA. We observed the interaction between PKCε and ERK by co-immunoprecipitation experiments. These observations suggest that PKCε activation contributes to the regulation of ERK1 activation. Bradykinin stimulated the accumulation of phosphorylated ERK in the nuclear fraction, that was inhibited in the cells treated with PKC inhibitors or transfected with PKCε siRNA. Consequently, we concluded that bradykinin activates PKCε via the PLD/PDK-1 pathway, which subsequently induces activation and translocation of ERK1 into the nucleus, and contributes to COX-2 expression for prostaglandin E_2_ synthesis in dermal fibroblasts.

## Introduction

Cyclooxygenase (COX) is the rate-limiting enzyme that catalyzes formation of prostanoids from arachidonic acid released from membrane phospholipids by phospholipase A_2_. There are two COX isoforms: COX-1 and COX-2. COX-1 is constitutively expressed for basal level as well as for immediate prostaglandin synthesis upon stimulation, particularly at high arachidonic acid concentrations. COX-2 is induced by cytokines and growth factors, and thus contributes to the inflammatory states^1–3^.

Protein kinase Cs (PKCs) represent a family of serine/threonine kinases that are implicated in various physiological and pathophysiological functions, including inflammation^4–8^. PKC isoforms are segregated into three subfamilies based on their homology and cofactor requirements for their activation. The conventional PKCs (cPKCs), PKCα, βI, βII, and γ, are activated by Ca^2+^ and diacylglycerol (DAG) in the presence of phosphatidylserine. The novel PKCs (nPKCs), PKCδ, ε, η, and θ, are activated by DAG in the presence of phosphatidylserine independent of Ca^2+^. The atypical PKCs (aPKCs), PKCζ and ι/λ, are activated in a Ca^2+^- and DAG-independent manner. cPKCs and nPKCs respond to the tumor-promoting phorbol esters, but aPKCs do not^4,6,7^. For the activation of PKCs, translocation of the enzymes from the cytosol to the plasma membrane is commonly observed^9^. Translocation of PKCs to cell compartments other than the plasma membrane, such as the nucleus and Golgi apparatus, has also been well documented^6,10,11^.

The mitogen-activated protein kinase (MAPK) signaling pathways are involved in the regulation of various cellular functions^12,13^. MAPKs are serine-threonine kinases that include extracellular signal-regulated kinase 1/2 (ERK1/2), c-Jun NH_2_-terminal kinase (JNK), p38 MAPK, and ERK5. MAPKs are usually localized in the cytoplasm of resting cells. After stimulation, activated-MAPKs translocate to different cellular compartments, including the nucleus. Subsequently, MAPKs phosphorylate a large number of transcription factors and thereby activate them to induce various physiological processes^14–16^.

Bradykinin, a nonapeptide formed by kallikrein-induced proteolytic cleavage of its precursor, a high-molecular-weight kininogen, is a potent inflammatory mediator. Bradykinin is implicated in the pathogenesis of inflammation, which induces pain, vasodilation, and an increase in vascular permeability^17^. Bradykinin has previously been reported to induce COX-2 expression in various cell types^18–21^, and PKC^22–24^ and MAPK^23,25,26^ have been considered to be involved in bradykinin-induced COX-2 expression. It has been reported that the nPKC isoform PKCε contributes to bradykinin-induced COX-2 expression in human airway smooth muscle cells^22^. In human airway epithelial cells and rat aortic vascular smooth muscle cells, ERK1/2 MAPK signaling pathway has been reported to be involved in bradykinin-induced COX-2 expression^25,26^. Additionally, in rat astrocytes, the contribution of PKCδ-dependent ERK1/2 activation to bradykinin-induced COX-2 expression has been investigated^23^.

In this study, we investigated bradykinin-induced COX-2 expression, which led to the synthesis of prostaglandin E_2_ in dermal fibroblasts. Our study found that bradykinin induces PKCε activation, leading to the activation and nuclear translocation of ERK1, and ultimately leads to COX-2 expression.

## Results

### Bradykinin-induced prostaglandin E_2_ and COX-2 expression via bradykinin 2 receptor and Gαq in dermal fibroblasts

**Figure 1:**
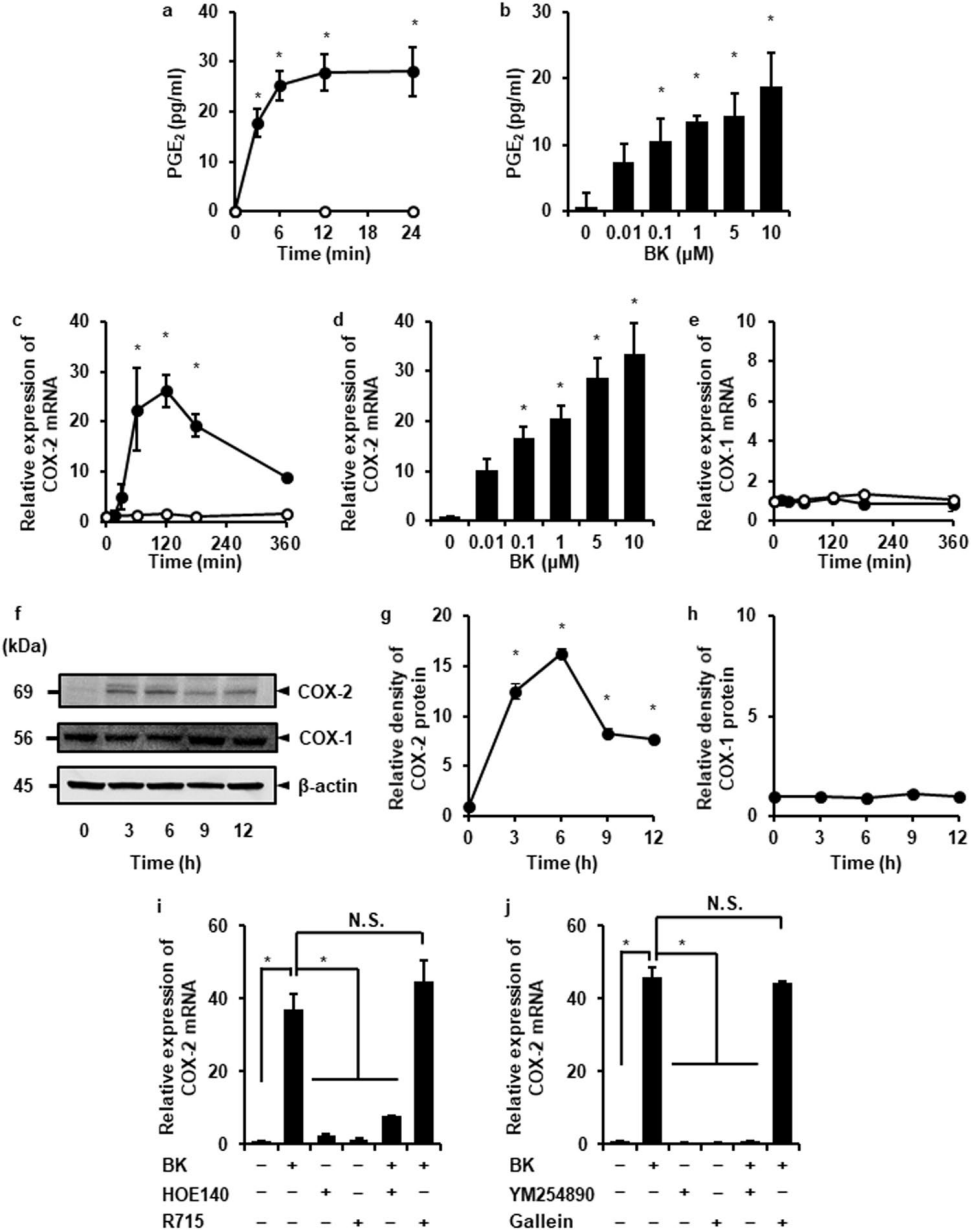
Bradykinin induces prostaglandin E_2_ and COX-2 expression in dermal fibroblasts via B2R and Gαq. (**a**,**b**) After the treatment with (closed circle) or without (open circle) 1 μM bradykinin (BK) for indicated time periods (**a**), or with indicated concentrations of BK for 12 h (**b**), prostaglandin E_2_ (PGE_2_) release was increased in a time- and dose-dependent manner. (**c–e**) In the cells treated with (closed circle) or without (open circle) 1 μM BK for indicated time periods (**c**), or with indicated concentrations of BK for 120 min (**d**), mRNA expression of COX-2 increased in a time- and dose-dependent manner, whereas BK had no effect on COX-1 mRNA expression (**e**). (**f–h**) In the cells treated with BK (1 μM), COX-2, COX-1, and β-actin (an internal standard), protein expression was examined (**f**). Relative density of COX-2 expression was altered in a time-dependent manner (**g**) but not that of COX-1 (**h**). (**i**) After the pretreatment with the B2R antagonist HOE140 (5 μM, 1 min) or the B1R antagonist R715 (1 μM, 5 min), the cells were incubated with 1 μM BK for 120 min. The B2R antagonist attenuated the BK-induced COX-2 mRNA expression but not the B1R antagonist. (**j**) After the pretreatment with the Gαq inhibitor YM254890 (100 nM, 30 min) or the Gβγ inhibitor Gallein (100 μM, 10 min), the cells were incubated with 1 μM BK for 120 min. The Gαq inhibitor attenuated the BK-induced COX-2 mRNA expression but not the Gβγ inhibitor. Results are presented as mean ± SE from 3 independent experiments. The F values were 44.81 (**a**), 10.30 (**b**), 19.18 (**c**), 27.37 (**d**), 172.79 (**g**), 49.50 (**i**), and 380.88 (**h**). The degrees of freedom were 7 (**a**), 5 (**b**), 6 (**c**), 5 (**d**), 4 (**g**), 5 (**i**), and 5 (**h**). *P < 0.05, compared with 0 h (**a**,**c**,**g**,**l**) or 0 pM (**b**,**d**).

In various kinds of cells (e.g., colonic myofibroblasts, gingival fibroblasts, and synovial fibroblasts), bradykinin induces prostaglandin E_2_ release^24,27–29^. Therefore, we first characterized bradykinin-induced prostaglandin E_2_ release in canine dermal fibroblasts. Bradykinin (1 μM) stimulated prostaglandin E_2_ release in a time-dependent manner (Fig. 1a). When the cells were treated with 0–10 μM bradykinin for 24 h, prostaglandin E_2_ release increased in a dose-dependent manner (Fig. 1b). Prostaglandin E_2_ synthesis was mediated by two COX isoforms, COX-1 and -2, which are constitutive and inducible forms, respectively^1,2^. Then, we examined the effect of bradykinin on the mRNA expression of COX isoforms. Bradykinin induced COX-2 mRNA expression in a time- and dose-dependent manner (Fig. 1c,d); however, it had no effect on COX-1 mRNA expression (Fig. 1e). Bradykinin also induced COX-2 protein expression in a time-dependent manner (Fig. 1f,g); however, the protein expression of COX-1 remained unaffected (Fig. 1f,h). The bradykinin 2 receptor (B2R) antagonist HOE140 and the trimeric G-protein Gαq inhibitor, YM254890, attenuated the bradykinin-induced COX-2 mRNA expression, whereas the bradykinin 1 receptor (B1R) antagonist R715 and the Gβγ inhibitor gallein had no effect (Fig. 1i,j). Taken together, these data confirm that bradykinin stimulates prostaglandin E_2_ release and COX-2 expression via B2R and Gαq in dermal fibroblasts.

### Contribution of PKCε in bradykinin-induced COX-2 mRNA expression

**Figure 2:**
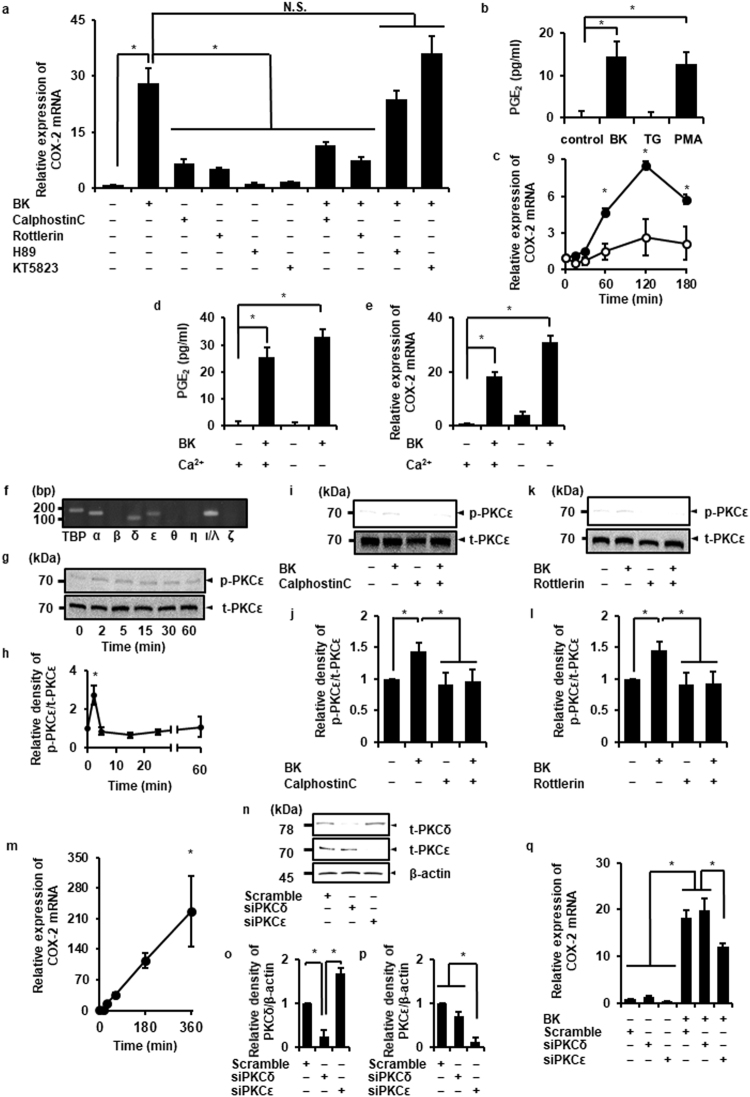
The contribution of PKCε to bradykinin-induced COX-2 expression. (**a**) After pretreatment with the pan-PKC inhibitor Calphostin C (1 µM, 30 min), the nPKC inhibitor Rottlerin (50 μM, 10 min), the PKA inhibitor H89 (10 μM, 60 min), or the PKG inhibitor KT5823 (1 μM, 60 min), cells were treated with bradykinin (BK; 1 μM) for 120 min. The pan- and nPKC inhibitors attenuated BK-induced COX-2 mRNA expression but not the PKA and PKG inhibitors. (**b**) Effect of phorbol 12-myristate 13-acetate (PMA; 100 nM, 12 h) and thapsigargin (TG; 5 μM, 12 h) on prostaglandin E_2_ (PGE_2_) release in dermal fibroblasts. PMA significantly provoked PGE_2_ release similar to BK, whereas TG had no effect. (**c**) PMA (closed circle; 100 nM) significantly induced COX-2 mRNA expression but not TG mRNA expression (open circle; 5 μM). (**d**,**e**) Removal of extracellular Ca^2+^ from the culture media had no effect on BK-induced PGE_2_ release (**d**) and COX-2 mRNA expression (**e**). (**f**) Expression of different subtypes of PKC was determined by RT-PCR using total RNA extracted from the dermal fibroblasts. PCR products for PKCα, δ, ε, and ι/λ were detected. TBP control was included in PCR as an internal standard. (**g**,**h**) When the cells treated with BK (1 μM), the expression of phosphorylated-PKCε (p-PKCε), total-PKCε (t-PKCε) was examined. Relative density of p-PKCε increased in a time-dependent manner (**h**,**i–l**) After pretreatment with pan-PKC inhibitor or the nPKC inhibitor, the cells were stimulated with BK (1 µM) for 2 min. The pan- and nPKC inhibitors attenuated the BK-induced phosphorylation of PKCε. Relative density of p-PKCε is illustrated (**j**,**l**,**m**) In the cells treated with the PKCε activator FR236924 (100 μM) for the indicated time periods, COX-2 mRNA expression was increased in a time-dependent manner. (**n–p**) PKCδ or ε siRNA-transfection resulted in a significant decrease of protein expression of PKCδ or ε protein, respectively, but not scramble siRNA-transfection (**n**). Relative density of PKCδ (**o**) or ε (**p**) protein expression in siRNA-transfected cells compared to that in scramble siRNA-transfected cells is illustrated. β-actin was used as an internal standard (**h–p**). (**q**) PKCε siRNA-transfection clearly inhibited the BK-induced COX-2 mRNA expression compared with PKCδ or scramble siRNA transfection. The F values were 38.22 (**a**), 34.80 (**b**), 235.06 (**c**), 58.47 (**d**), 116.69 (**e**), 7.00 (**h**), 7.80 (**j**), 10.36 (**l**), 7.99 (**m**), 31.29 (**o**), 45.57 (**p**), and 56.52 (**q**). The degrees of freedom were 9 (**a**), 3 (**b**), 5 (**c**), 3 (**d**), 3 (**e**), 5 (**h**), 3 (**j**), 3 (**l**), 2 (**o**), 2 (**p**), and 5 (**q**). *P < 0.05, compared with 0 h (**c**,**h**,**m**).

B2R transduces extracellular signals through the activation of G-proteins and AGC protein kinases (i.e. PKA, PKG, and PKC)^30,31^. Next, we examined the effect of the AGC protein kinase inhibitors on bradykinin-induced COX-2 mRNA expression. In the presence of the pan-PKC inhibitor Calphostin C or the nPKC inhibitor Rottlerin bradykinin failed to induce COX-2 mRNA expression, whereas PKA and PKG inhibitors, H89 and KT5823, respectively, had no effect (Fig. 2a).

Conventional PKC (α, β) and novel PKC (δ, ε, θ, η), but not atypical PKC (ι/λ, ζ), require DAG for the activation. The functional analog phorbol 12-myristate 13-acetate (PMA) activates the PKCs by binding to the DAG binding site of the PKCs with high affinity. In dermal fibroblasts, PMA induced prostaglandin E_2_ release and COX-2 mRNA expression (Fig. 2b,c), suggesting that PKCs activated by DAG are implicated in COX-2 expression. Bradykinin has been reported to induce an increase in intracellular Ca^2+^ concentrations ([Ca^2+^]_i_)^32–34^. Then, we examined the contribution of Ca^2+^ mobilization to bradykinin-induced COX-2 expression in dermal fibroblasts. Bradykinin induced a transient increase in [Ca^2+^]_i_ in the presence of extracellular Ca^2+^, and the effect was reduced in the absence of extracellular Ca^2+^ (Supplementary Fig. 2a). However, removal of extracellular Ca^2+^ from culture media had no effect on the bradykinin-induced prostaglandin E_2_ release (Fig. 2d) and COX-2 mRNA expression (Fig. 2e). The Ca^2+^ pump inhibitor thapsigargin induced an increase in [Ca^2+^]_i_ (Supplementary Fig. 2b), but failed to stimulate prostaglandin E_2_ (Fig. 2b) and COX-2 mRNA expression (Fig. 2c). These observations suggest that Rottlerin- and PMA-sensitive and Ca^2+^-independent PKC is involved in bradykinin-induced COX-2 mRNA expression.

In dermal fibroblasts, mRNA expression of PKCα, δ, ε, and ι/λ was detected (Fig. 2f). Among these, PKCδ and ε are PMA-sensitive and Ca^2+^-independent^4,6,7^. Rottlerin has been used as a specific inhibitor of PKCδ^35^, but recent studies reported that rottlerin also inhibited other kinases including PKCε^36,37^. Therefore, we examined the effect of bradykinin on phosphorylation of PKCδ and ε. In the cells stimulated with bradykinin, the phosphorylation of PKCε occurred in a time-dependent manner (Fig. 2g,h), but not that of PKCα, δ, and ι/λ (Supplementary Fig. 2c–e). The pan-PKC inhibitor and Rottlerin attenuated the bradykinin-induced PKCε phosphorylation (Fig. 2i–l). Moreover, we treated the cells with the PKCε activator, FR236924. As shown in Fig. 2m, the PKCε activator stimulated the COX-2 mRNA expression in a time-dependent manner. Antisense techniques or siRNA transfection has been implemented specifically to inhibit PKC subtypes. Therefore, we performed PKCε knockdown experiment using siRNA transfection. As shown in Fig. 2n–p, the mRNA and protein expression of PKCδ or ε was significantly inhibited by the transfection with respective siRNAs, but not with scramble siRNA as a control. Bradykinin-induced COX-2 mRNA expression was inhibited in the PKCε siRNA-transfected cells compared with the scramble or PKCδ siRNA-transfected cells (Fig. 2q). Taken together, our results strongly suggest that PKCε contributes to the bradykinin-induced COX-2 expression.

### Contribution of PLD/PDK-1 signaling to bradykinin-induced COX-2 expression via PKCε activation

**Figure 3:**
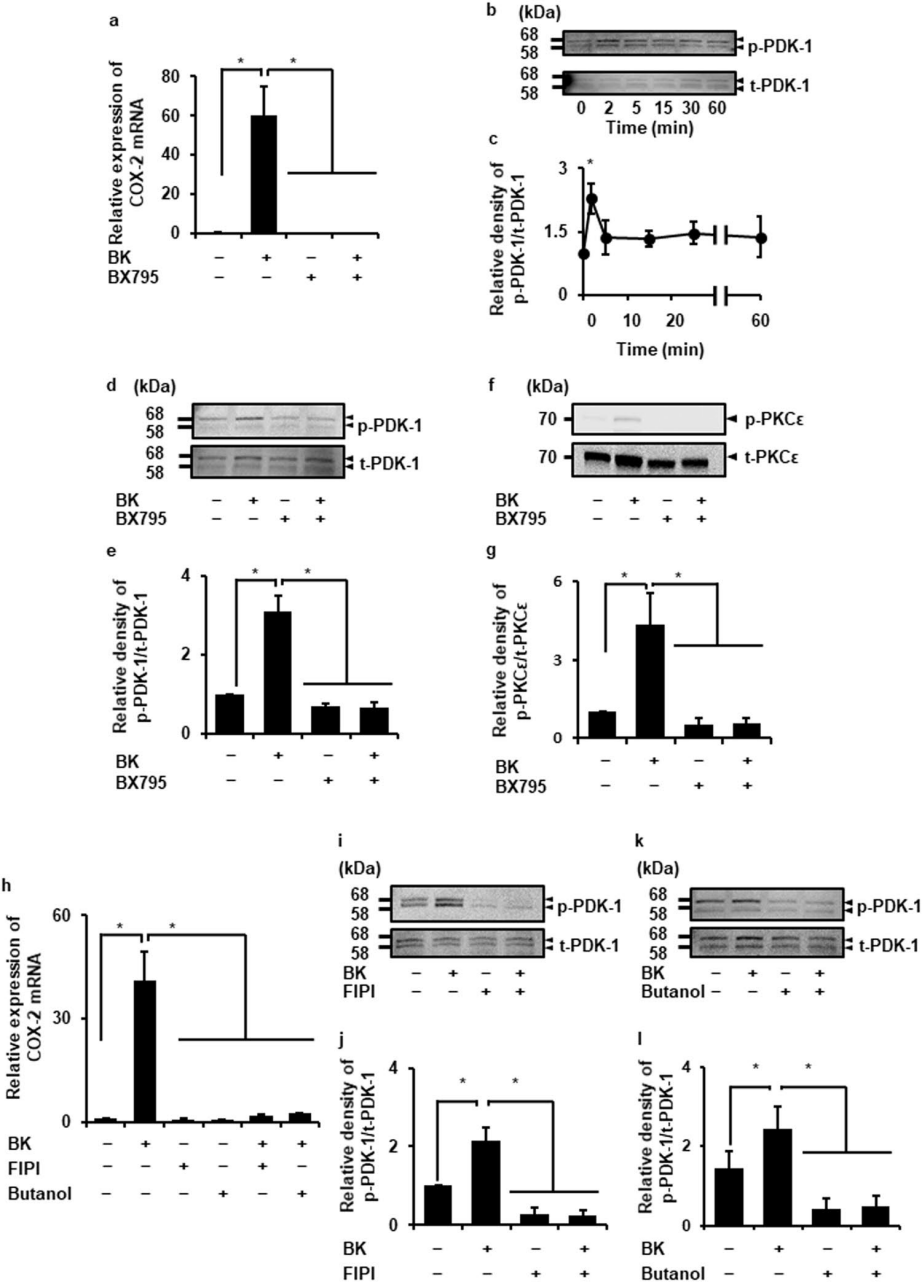
PLD/PDK-1 signaling contributes to bradykinin-induced COX-2 expression via PKCε. (**a**) After the pretreatment with the PDK-1 inhibitor BX795 (30 µM, 1 h), the cells were stimulated with bradykinin (BK; 1 µM) for 120 min. The inhibitor for PDK-1 clearly attenuated the BK-induced COX-2 mRNA expression. (**b**,**c**) The expression of phosphorylated PDK-1 (p-PDK-1) and total PDK-1 (t-PDK-1) in the cells stimulated with BK (1 μM) (**b**). Relative density of p-PDK-1 increased in a time-dependent manner (**c**). (**d–g**) After the pretreatment with the PDK-1 inhibitor BX795 (30 µM, 1 h), the cells were stimulated with BK (1 µM) for 2 min. The BK-induced phosphorylation of PDK-1 (**d**) and PKCε (p-PKCε; **g**) was inhibited by the PDK-1 inhibitor. Relative density of p-PDK-1 (**d**) and p-PKCε (**g**) compared with that in the absence of BK is shown. (**h**) After the pretreatment with the small molecule PLD inhibitor FIPI (50 µM, 1 h) or the metabolic PLD inhibitor butanol (1%, 30 min), the cells were stimulated with BK (1 µM) for 120 min. The inhibitors for PLD clearly attenuated the BK-induced COX-2 mRNA expression. (**i-l**) BK (1 μM, 2 min) failed to induce the expression of p-PDK-1 in the cells pretreated with FIPI (**i**,**j**) or butanol (**k**,**l**). Relative density of p-PDK-1 (**j**,**l**) compared with that in the absence of BK is shown. Results are presented as mean ± SE from 3 independent experiments. The F values were 15.97 (**a**), 6.16 (**c**), 26.88 (**e**), 8.16 (**g**), 23.32 (**h**), 30.95 (**j**), and 28.56 (**l**). The degrees of freedom were 3 (**a**), 5 (**c**), 3 (**e**), 3 (**g**), 5 (**h**), 3 (**j**), and 3 (**l**). *P < 0.05, compared with 0 h (**c**).

It has been reported that 3-phosphoinositide-dependent protein kinase-1 (PDK-1), a serine/threonine kinase, is involved in the activation of PKC isoforms including PKCε^38^. As show in Fig. 3a, the PDK-1 inhibitor, BX795, attenuated bradykinin-induced COX-2 mRNA expression. Bradykinin induced the phosphorylation of PDK-1, which is attenuated by the PDK-1 inhibitor (Fig. 3b–e). We observed that bradykinin failed to induce the phosphorylation of PKCε in the presence of the PDK-1 inhibitor (Fig. 3f,g), suggesting that PDK-1 contributed to bradykinin-induced PKCε activation, and subsequently induced COX-2 mRNA expression.

PKCε is activated by diacylglycerol (DAG). The intracellular DAG levels are upregulated by the activation of phospholipase C (PLC), phospholipase D (PLD), or phosphatidylinositol 3,4,5-trisphosphate (PI3K)^39–43^. We investigated the effect of the PLC, PLD, or PI3K inhibitor on the bradykinin-induced COX-2 mRNA expression. Although the PLC inhibitor U73122, pan-PI3K inhibitor LY294002, PI3Kβ inhibitor TGX221, and PI3Kγ inhibitor AS605240 failed to inhibit the bradykinin-induced COX-2 mRNA expression (Supplementary Fig. 3), the PLD inhibitors butanol and FIPI clearly attenuated bradykinin-induced COX-2 mRNA expression (Fig. 3h). Considering the outcomes of inhibitor experiments, it is likely that PLD contributes to COX-2 expression in dermal fibroblasts stimulated with bradykinin. The PLD inhibitors attenuated bradykinin-induced phosphorylation of PDK-1 (Fig. 3i–l). These observations suggest that PLD/PDK-1 signaling contributes to bradykinin-induced activation of PKCε and subsequently induces COX-2 expression.

### Involvement of ERK1 in bradykinin-induced COX-2 mRNA expression

**Figure 4:**
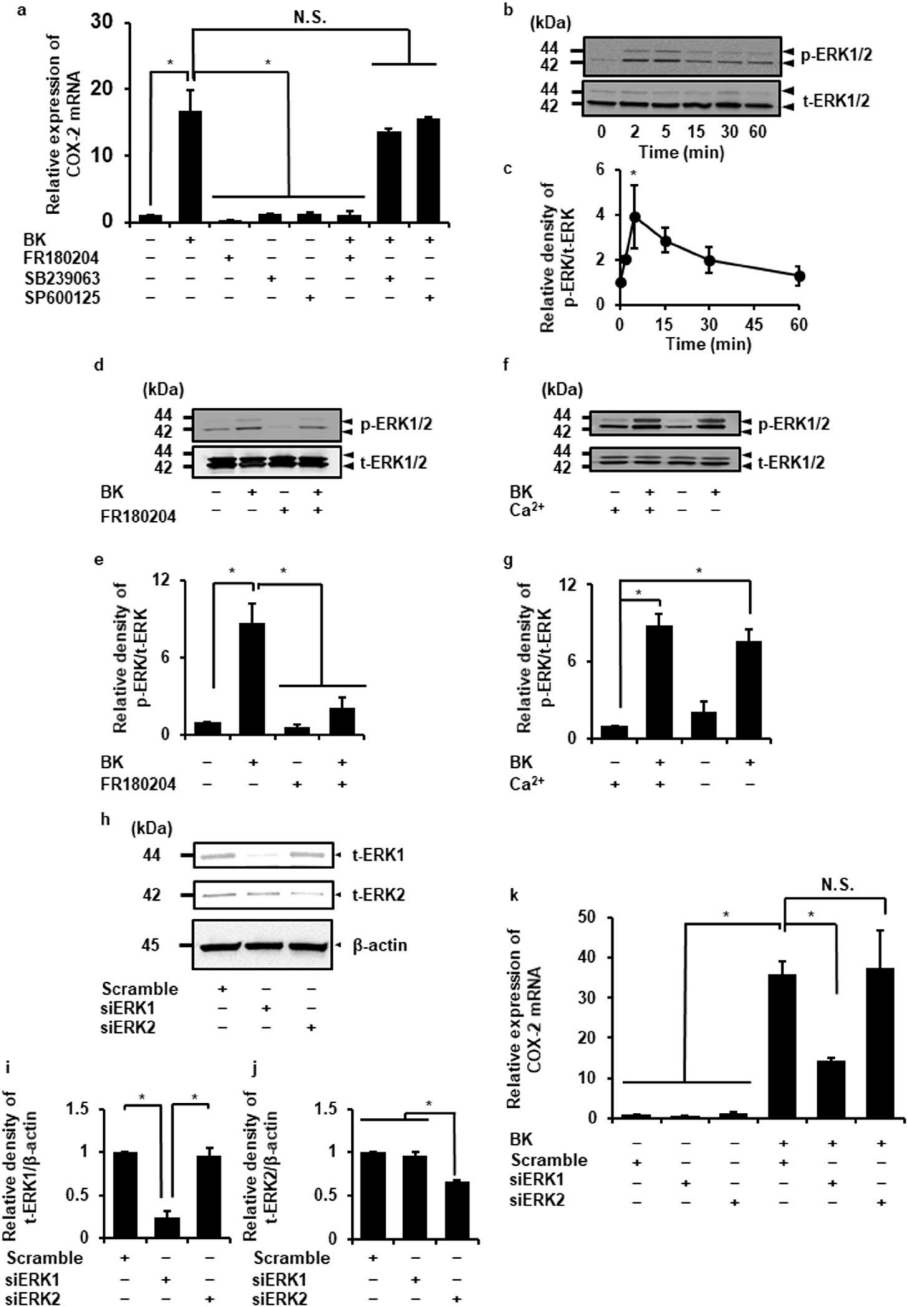
The contribution of MAPK on bradykinin-induced COX-2 expression. (**a**) After the pretreatment with the ERK inhibitor FR180204 (50 µM), the p-38 inhibitor SB239063 (20 µM), or the JNK inhibitor SP600125 (10 µM) for 60 min, the cells were stimulated with bradykinin (BK, 1 µM) for 120 min. The ERK inhibitor significantly attenuated the BK-induced COX-2 mRNA expression, whereas the p-38 inhibitor and the JNK inhibitor had no effect. (**b**,**c**) In the cells treated with BK (1 μM) for 0–60 min, the expression of phosphorylated ERK1/2 (p-ERK) and total ERK1/2 (t-ERK) was examined (**b**). Relative density of p-ERK1/2 was altered in a time-dependent manner (**c**). (**d**,**e**) After pretreatment with the ERK inhibitor (50 μM, 1 h), the cells were stimulated with BK (1 μM) for 5 min. The ERK inhibitor attenuated the BK-induced phosphorylation of ERK1/2. Relative density of p-ERK1/2 was illustrated (**e**). (**f**,**g**) The removal of extracellular Ca^2+^ from the culture media had no effect on the BK-induced ERK1/2 phosphorylation. Relative density of p-ERK1/2 was illustrated (**g**). (**h**) ERK1 or ERK2 siRNA-transfection resulted in a significant decrease of expression of t-ERK1 or t-ERK2 protein, respectively, but not with scramble siRNA transfection. β-actin was used as an internal standard. (**i**,**j**) Relative expression of ERK1 (**i**) or ERK2 protein (**j**) in siRNA-transfected cells compared to that in scramble siRNA transfected cells is illustrated. (**k**) ERK1 siRNA transfection clearly inhibited the BK-induced COX-2 mRNA expression compared with ERK2 or scramble siRNA transfection. Results are presented as mean ± SE from 3 independent experiments. The F values were 6.88 (**a**), 3.42 (**c**), 14.00 (**e**), 25.13 (**g**), 76.60 (**i**), 107.17 (**j**), and 21.41 (**k**). The degrees of freedom were 7 (**a**), 5 (**c**), 3 (**e**),3 (**g**), 2 (**i**), 2 (**j**), and 7 (**k**). *P < 0.05, compared with 0 h (**c**).

In mammalian cells, MAPK signaling plays a crucial role in inflammatory responses. Three major MAPK signaling pathways, ERK, JNK, and p38 MAPK, have been demonstrated. We examined the contribution of MAPK signaling pathways to bradykinin-induced COX-2 expression in dermal fibroblasts by MAPK inhibitors. The ERK inhibitor, FR180204, clearly inhibited bradykinin-induced COX-2 mRNA expression, but not p38 and JNK inhibitors, SB239063 and SP600125, respectively (Fig. 4a).

Next, we investigated the effect of bradykinin on the phosphorylation of ERK. In cells stimulated with bradykinin, ERK phosphorylation occurred in a time-dependent manner. The peak of phosphorylation was observed after 5 min of bradykinin treatment (Fig. 4b,c). The ERK inhibitor attenuated the bradykinin-induced ERK phosphorylation (Fig. 4d,e). The removal of extracellular Ca^2+^ had no effect on the bradykinin-induced ERK phosphorylation (Fig. 4f,g). The inhibitors for up-stream components (*e*.*g*. B2R, PDK-1, and PLD) also inhibited the bradykinin-induced phosphorylation of ERK (Supplementary Fig. 4). To confirm the involvement of ERK in bradykinin-induced COX-2 mRNA expression, we performed knockdown experiment using siRNA transfection. As shown in Fig. 4h–j, ERK1 and 2 protein expression was significantly reduced by transfection with respective siRNAs, but not by scramble siRNA transfection. Bradykinin-induced COX-2 mRNA expression was attenuated in the ERK1 siRNA-transfected cells compared to that in scramble or ERK2 siRNA-transfected cells (Fig. 4k). These results strongly suggest that ERK signaling pathway, especially ERK1, is involved in the bradykinin-induced COX-2 expression in dermal fibroblasts.

### Interaction of PKCε and ERK signaling in bradykinin-stimulated dermal fibroblasts

**Figure 5:**
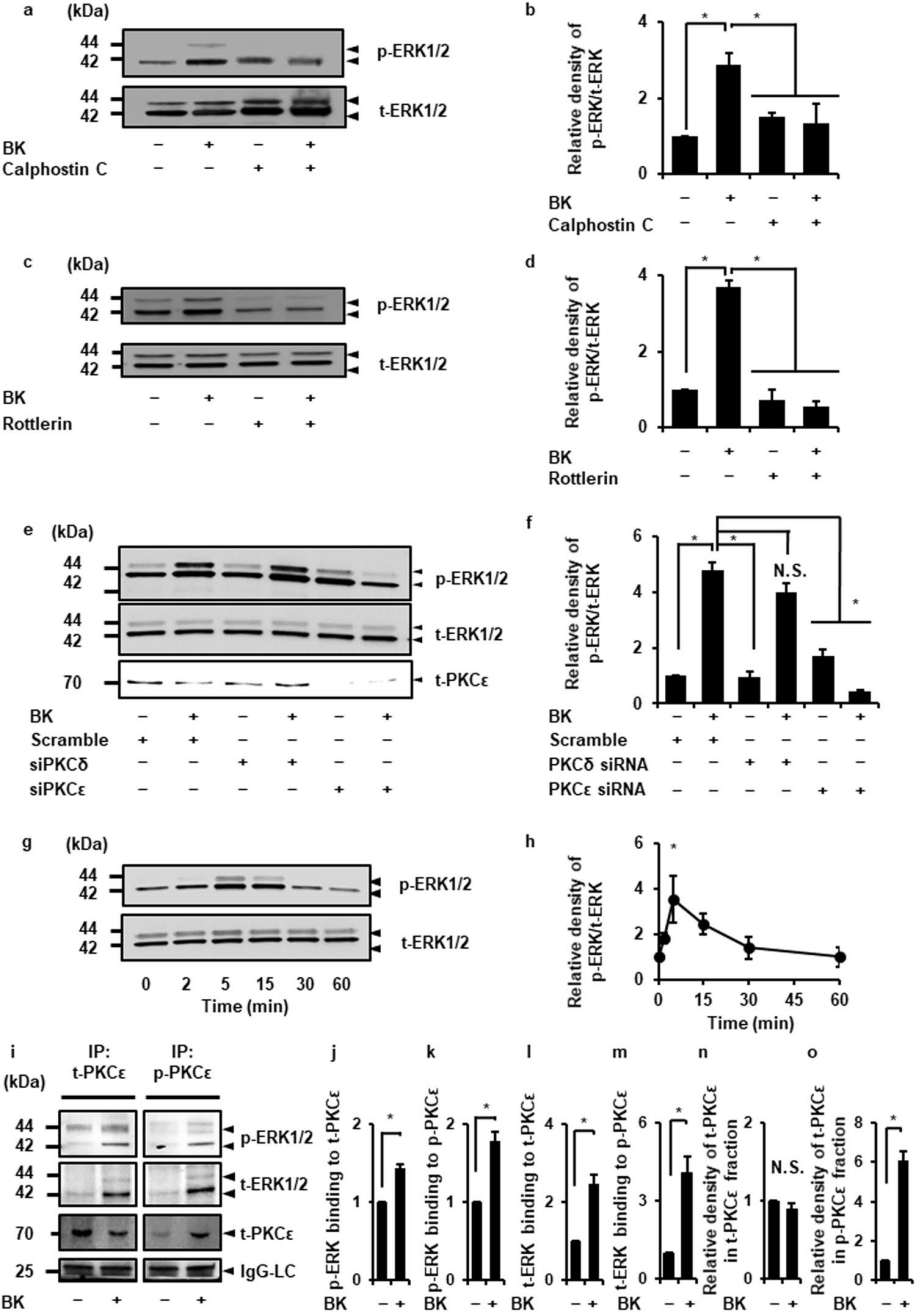
PKCε contributes to bradykinin-induced ERK activation. (**a**) After the pretreatment with the pan-PKC inhibitor Calphostin C (1 µM, 30 min) or the nPKC inhibitor Rottlerin (50 μM, 10 min), the cells were stimulated with bradykinin (BK, 1 µM) for 5 min. (**a–d**) The pan- and nPKC inhibitors attenuated BK-induced phosphorylation of ERK1/2 (p-ERK1/2; **a**,**c**). Relative density of p-ERK1/2 (**b**,**d**) compared with that in the absence of BK is shown. (**e**,**f**) PKCε siRNA transfection clearly attenuated the BK-induced ERK1/2 phosphorylation compared with PKCδ or scramble siRNA transfection (**e**). Relative density of p-ERK1/2 compared with that in the absence of BK is shown (**f**,**g**,**h**) When the cells were treated with the PKCε activator FR236924 (100 μM) for 60 min, the expression of p-ERK1/2 increased in a time-dependent manner (**g**). Relative density of p-ERK1/2 compared with that in the absence of PKCε activator is shown (**h**). (**i**, **j**) In the fibroblasts stimulated with or without BK (1 μM) for 5 min, the fractions immunoprecipitated with anti-total-PKCε (t-PKCε) or anti-phosphorylated-PKCε (p-PKCε) antibodies were isolated. The levels of p-ERK1/2, t-ERK1/2, or t-PKCε were detected by western blotting (**i**). Relative density of p-ERK in the fractions precipitated with anti-t-PKCε (**j**) or anti-p-PKCε (**k**) antibodies compared with that in the absence of BK is shown. Relative density of t-ERK in the fractions precipitated with anti-t-PKCε (**l**) or anti-p-PKCε (**m**) antibodies compared with that in the absence of BK is shown. Relative density of t-PKCε in the fractions precipitated with anti-t-PKCε (**n**) or anti-p-PKCε (**o**) antibodies compared with that in the absence of BK is shown. For immunoblotting, total cell lysate (100 μg protein) was used. IgG light chain (LC) were used as a loading control. There is no significant difference in IgG-LC between control and BK-stimulated cells. Results are presented as mean ± SE from 3 independent experiments. The F values were 9.26 (**b**), 68.82 (**d**), 70.45 (**f**), and 5.94 (**h**). The t values were 9.43 (**j**), 7.04 (**k**), 6.22 (**l**), 5.27 (**m**) and 11.6 (**o**). The degrees of freedom were 3 (**b**), 3 (**d**), 5 (**f**), 5 (**h**), and 2 (**j**), 2 (**k**), 2 (**l**), 2 (**m**) and 2 (**o**). *P < 0.05, compared with 0 h (**h**).

To elucidate the relationship between PKCε and ERK signaling, the effect of PKC inhibitors, Calphostin C and Rottlerin, on the bradykinin-induced ERK phosphorylation was examined. As shown in Fig. 5a–d, both PKC inhibitors attenuated bradykinin-induced ERK phosphorylation, suggesting that PKCε evokes ERK activation and subsequently induces COX-2 expression in bradykinin-stimulated cells. To confirm our hypothesis regarding the crucial role of PKCε in the activation of the ERK signaling pathway, we performed PKCε knockdown experiments by siRNA transfection. Bradykinin-induced phosphorylation of ERK was clearly inhibited in the fibroblasts transfected with PKCε siRNA, but not in those transfected with scramble or PKCδ siRNA (Fig. 5e,f). In the cells treated with the PKCε activator, FR236924, the phosphorylation of ERK and PKCε occurred in a time-dependent manner (Fig. 5g,h, Supplementary Fig. 5). In co-immunoprecipitation experiments, the total PKCε level in the fraction precipitated with anti-phospho-PKCε antibody was increased (Fig. 5i,o), whereas that with anti-total-PKCε antibody remained unchanged (Fig. 5i,n). The levels of total and phosphorylated ERK expression were increased in the fractions precipitated with anti-total and anti-phospho-PKCε antibodies (Fig. 5i–m). Taken together, our results indicate that PKCε plays a crucial role in ERK1 activation in bradykinin-stimulated cells.

### Regulation of PKCε and ERK accumulation in the nuclei upon bradykinin stimulation

**Figure 6:**
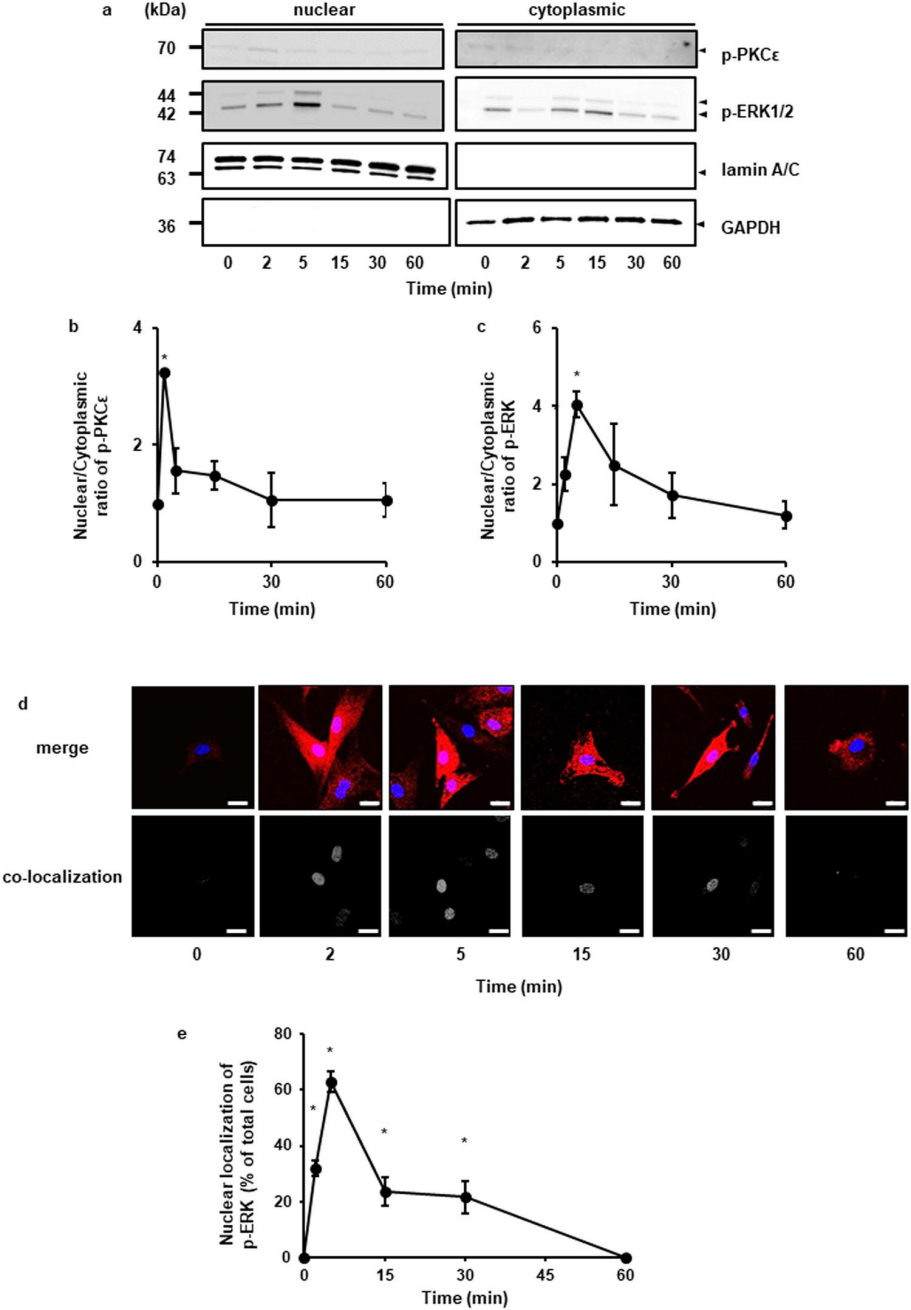
Localization of phosphorylated ERK1/2 in bradykinin-stimulated dermal fibroblasts. (**a–c**) After the treatment with bradykinin (BK, 1 μM) for the indicated time periods, the nuclear and cytoplasmic fractions of the cells were isolated and subjected to western blot analysis. Lamin A/C and GAPDH served as nuclear and cytoplasmic markers, respectively. Nuclear accumulation of phosphorylated PKCε (p-PKCε) and phosphorylated ERK1/2 (p-ERK1/2) increased in a time-dependent manner, which correlated with a concomitant decrease in the cytoplasmic levels (**a**). Relative density of nuclear/cytoplasmic ratio of p-PKCε (**b**) and p-ERK1/2 (**c**) is shown. (**d**) Cells were stimulated with BK (1 μM) for the indicated time periods and were labeled with fluorescent dye for nuclear staining (blue, upper panels) and antibodies against p-ERK1/2 (red, upper panels). The lower panels show colocalized regions as white dots using the colocalization analysis plug-in embedded in BioImage XD. (**e**) The percentage of cells detected nuclear translocation of p-ERK1/2 is shown. The F values were 6.63 (**b**), 5.72 (**c**), and 33.11 (**e**). The degrees of freedom were 5 (**b**), 5 (**c**), and 5 (**e**). *P < 0.05, compared with 0 h (**b**,**c**,**e**).

**Figure 7:**
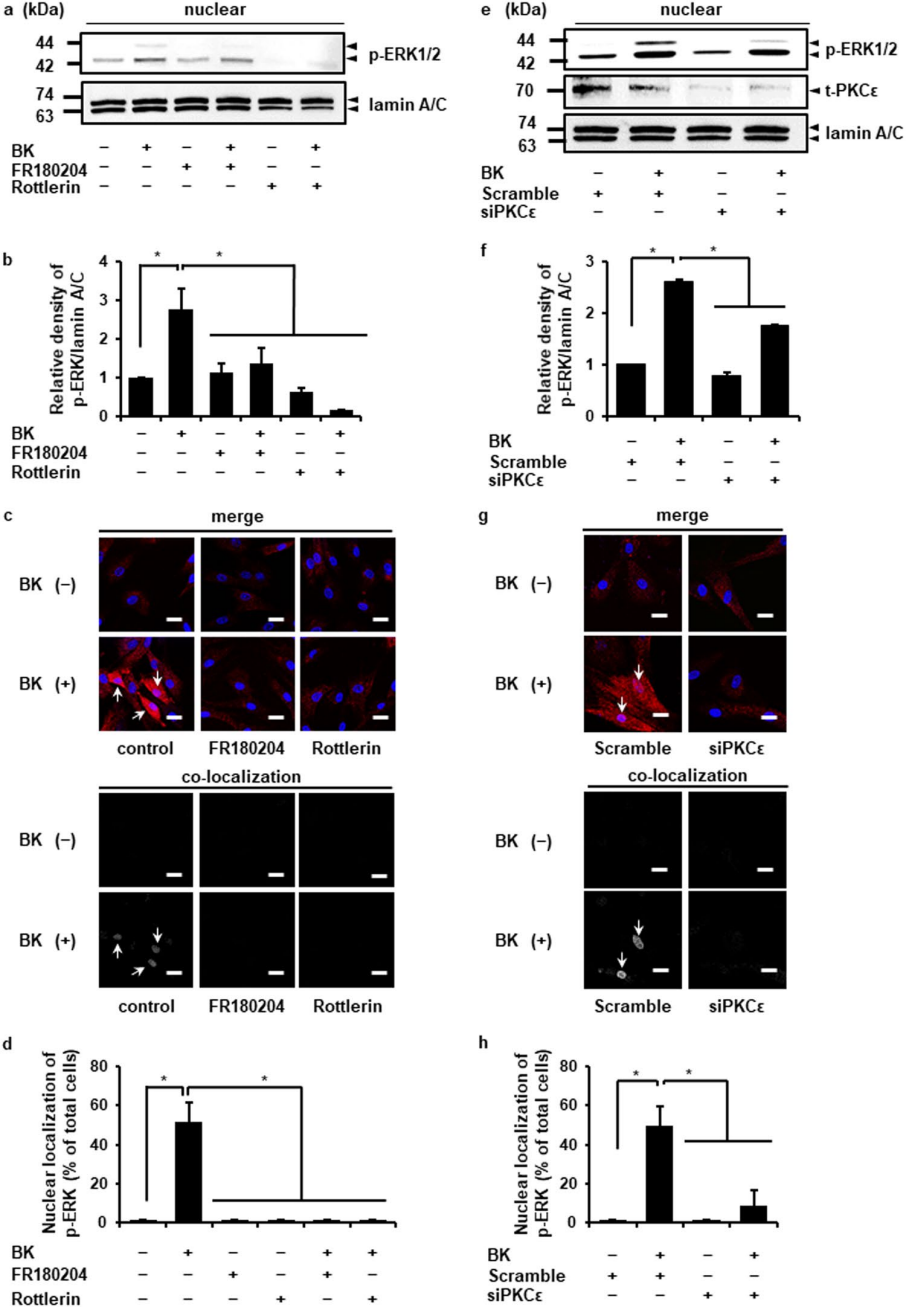
PKCε modulates the bradykinin-induced nuclear accumulation of p-ERK1/2. The nuclear fractions isolated from the cells stimulated with bradykinin (BK) were subjected to western blot analysis (**a**,**e**). Relative density of p-ERK1/2 in the nuclear fraction is shown (**b**,**f**). The fixed cells were labeled with fluorescent dye for nuclear staining (**c**,**g**; blue, upper panels) and antibodies against p-ERK1/2 (**c**,**g**; red, upper panels). The lower panels show colocalized regions as white dots using the colocalization analysis plug-in embedded in BioImage XD (**c**,**g**). The percentage of cells with nuclear translocation of p-ERK1/2 is shown (**d**,**h**). (**a–d**) After the pretreatment with the ERK inhibitor FR180204 (50 μM, 1 h) or the nPKC inhibitor Rottlerin (50 μM, 10 min), the cells were stimulated with BK (1 μM) for 5 min. The ERK and nPKC inhibitors attenuated the BK-induced nuclear accumulation of phosphorylated ERK1/2 (p-ERK1/2). (**e–h**) PKCε siRNA transfection clearly inhibited the BK-induced nuclear accumulation of p-ERK1/2 compared with scramble siRNA transfection. Results are presented as mean ± SE from 3 independent experiments. The F values were 17.22 (**b**), 28 (**d**), 1234.25 (**f**), and 11 (**h**). The degrees of freedom were 5 (**b**), 5 (**d**), 3 (**f**), and 3 (**h**). *P < 0.05.

Next, we examined the effect of bradykinin on the intracellular localization of phosphorylated-PKCε and phosphorylated-ERK by subcellular fractionation and immunocytochemical analysis. In the nuclear fraction of bradykinin-stimulated cells, the expression of phosphorylated-PKCε and -ERK transiently increased and the peak level was observed at 2 and 5 min after stimulation, respectively (Fig. 6a–c). As Fig. 6d,e summarize, the co-localization of phosphorylated-ERK1/2 and the nuclear marker TO-PRO-3 time-dependent stimulation with bradykinin treatment was confirmed by immunocytochemical study. Then, we investigated the effect of ERK inhibitor, FR180204, and nPKC inhibitor, Rottlerin, on the bradykinin-induced accumulation of phosphorylated-ERK in the nuclei. In the presence of the inhibitors of ERK and nPKC, bradykinin failed to induce the accumulation of phosphorylated-ERK in the nuclei (Fig. 7a–d). To confirm the contribution of PKCε on the bradykinin-induced accumulation of phosphorylated-ERK in the nuclei, we performed PKCε knockdown experiment using siRNA transfection. Bradykinin-induced accumulation of phosphorylated-ERK in the nuclei was attenuated in the PKCε siRNA-transfected cells compared to that in cells transfected with the scramble siRNA (Fig. 7e–h). These results indicate that bradykinin-induced ERK phosphorylation contributes to ERK translocation to the nuclei and PKCε regulates the bradykinin-induced nuclear translocation of phosphorylated-ERK.

## Discussion

In this study, we demonstrated that bradykinin induced the mRNA and protein expression of COX-2 and subsequently promoted prostaglandin E_2_ release from dermal fibroblasts. Bradykinin elicits various actions related to physiological and pathophysiological functions via two bradykinin receptor B1R and B2R. B2R is constitutively expressed in a variety of tissues, whereas B1R is expressed at low levels and induced by inflammation and injury^44^. The activation of these receptors stimulates PLC coupled to G-protein, which provokes the increase in [Ca^2+^] _i_ and DAG^45^. We observed that the pharmacological inhibitors B2R and Gαq completely attenuated the effect of bradykinin on COX-2 mRNA expression, strongly suggesting that bradykinin induces COX-2 expression via B2R coupled to Gαq in dermal fibroblasts.

Bradykinin has been demonstrated to stimulate COX-2 expression via PKC activation^22,23,26^. In this study, we demonstrated that bradykinin activates PKCε, which is independent of [Ca^2+^]_i_ and provoked by PMA-activation, in dermal fibroblasts. As bradykinin failed to induce COX-2 mRNA expression in the fibroblasts transfected with PKCε siRNA, we concluded that PKCε contributes to bradykinin-induced COX-2 expression and prostaglandin E_2_ release in dermal fibroblasts.

PKCε requires DAG for its activation^6,46^. DAG has been demonstrated to be generated by hydrolysis of phosphatidylinositol 4,5-bisphosphate (PIP_2_) by phospholipase C (PLC)^4^. However, a PLC inhibitor had no effect on bradykinin-induced COX-2 mRNA expression in dermal fibroblasts. Conversely, FIPI and butanol, a small molecule inhibitor and a metabolic inhibitor of phospholipase D (PLD), respectively, completely attenuated bradykinin-induced COX-2 mRNA expression. It has been reported that PLD hydrolyzes phosphatidylcholine to generate phosphatidic acid (PA) and phosphatidate phosphohydrolase sequentially dephosphorylates the PA to generate DAG^47,48^. DAG binds to the C1 domain of PKCε and regulates its activation^6,46^. These results suggest that PLD contributes to bradykinin-induced COX-2 mRNA expression via the generation of DAG in dermal fibroblasts.

We also demonstrated that the pharmacological inhibition of PDK-1 attenuated bradykinin-induced COX-2 mRNA expression in dermal fibroblasts. PDK-1 is known to be a critical upstream kinase to activate PKC isoforms, which phosphorylates a conserved threonine residue in the activation loop of PKC isoforms^49^. PDK-1 has been reported to phosphorylate PKCε, which leads to activation^50–53^. In dermal fibroblasts, bradykinin induced PDK-1 phosphorylation. The PDK-1 inhibitor, BX795, inhibited bradykinin-induced PDK-1 phosphorylation and PKCε phosphorylation. Furthermore, in the cells treated with the PDK-1 inhibitor, bradykinin failed to induce COX-2 mRNA expression. These observations suggest that PKCε activated by PDK-1 contributes to bradykinin-induced COX-2 mRNA expression.

As PI3K lipid products, such as phosphatidylinositol 3,4-bisphosphate and phosphatidylinositol 3,4,5-trisphosphate, bind and recruit PDK-1 to membranes leading to phosphorylation of downstream substrates including PKC isoforms, the phosphorylation by PDK-1 provides a link with the PI3K pathway^54^. A requirement of PI3K- and PDK-1-dependent phosphorylation of PKCε leading to its activation has been demonstrated^52^. However, PI3K inhibitors had no effect on bradykinin-induced COX-2 mRNA expression in dermal fibroblasts. On the other hand, interestingly, FIPI and butanol, a small molecule inhibitor and a metabolic inhibitor of PLD, respectively, completely attenuated bradykinin-induced PDK-1 phosphorylation and COX-2 mRNA expression. These results suggest that bradykinin activates PDK-1 via PLD, which subsequently activates PKCε, and consequently induces COX-2 mRNA expression. Although PLD appears to contribute to PKCε activation by the supply of DAG and through PDK activation, further studies are required to completely understand the mechanism.

Multiple MAPK signaling pathways coordinate and integrate responses from diverse stimuli including proinflammatory cytokines (e.g. IL-1β and TNF-α)^55,56^. However, the response of MAPK signaling is highly dependent on the cellular context. In this study, a pharmacological inhibitor of ERK completely attenuated bradykinin-induced COX-2 mRNA expression, suggesting that ERK activation is involved in bradykinin-induced COX-2 expression. In fact, bradykinin stimulated ERK phosphorylation in a time-dependent manner. ERK isoforms, ERK1 and ERK2, share 83% amino acid identity and are co-expressed in a variety of tissues^57,58^, ERK1 and ERK2 have been reported to be co-activated in response to several extracellular stimuli^59–61^. However, we previously reported the functional difference between the two isoforms in canine synovial fibroblasts^56^. Then we examined the effect of bradykinin on COX-2 mRNA in the cells transfected with ERK1 or ERK2 siRNA. Bradykinin-induced COX-2 mRNA expression was inhibited in ERK1-knock-down cells, but not in ERK2-knock-down cells. These observations indicate that bradykinin induces COX-2 expression via ERK1 activation in dermal fibroblasts.

We also demonstrated the relationship between PKCε and ERK signaling in bradykinin-treated cells. A PKCε activator stimulated ERK phosphorylation. Bradykinin-induced ERK phosphorylation was attenuated by the pharmacological inhibitors which inhibited bradykinin-stimulated PKCε activation, and in the PKCε-knockdown cells. Moreover, the binding of phosphorylated-PKCε and -ERK was observed in the cells stimulated with bradykinin. Taken together, it is most probable that PKCε is an upstream regulator for ERK activation in bradykinin-induced COX-2 expression. In general, ERK is considered to be phosphorylated by MEK, the upstream kinase MAPKK^12,13^. Therefore, further studies are needed to understand the relationship between PKCε, ERK, and MEK.

In this study, we demonstrated that bradykinin induced the nuclear localization of phosphorylated-ERK. In the PKCε knockdown cells, the effect of bradykinin on the nuclear localization of phosphorylated-ERK was attenuated. These results suggest that PKCε plays a crucial role in the bradykinin-induced nuclear localization of phosphorylated-ERK in fibroblasts. We also demonstrated that the activation of PKCε was induced by PDK-1. However, it is obscure how PDK-1, which is reportedly localized to the membrane, induces the activation of PKCε. Further work to study the interaction of PDK-1 and PKCε in dermal fibroblasts is being carried out in our laboratory. The nuclear translocation of ERK is considered to be an important event for the activation of transcription factors for translation of mRNA. The main activity of ERK in the nucleus seems to be the activation of transcription factors and binding to the DNA as transcription factor for some genes^62,63^. Although ERK does not contain the canonical nuclear localization signal, ligand-induced nuclear translocation of ERK has been reported^64–66^.

ERK contains two phosphorylation sites. The first is the threonine-glutamine-tyrosine (TEY) sequence, which is phosphorylated by MEK, an MAPKK, and is important for the release of ERK from its cytoplasmic anchors^67^. The second phosphorylation site is the serine-proline-serine (SPS) sequence for nuclear translocation^68^. SPS sequence contained two serine residues separated by a proline, when mutated to Alanine or deleted, prevented the translocation of ERK, suggesting that the phosphorylation of not only TEY motif but also SPS sequence is essential for the function of ERK^67–69^. In a previous study, Casein kinase 2, a ubiquitous protein serine/threonine kinase, has been reported to be involved in the nuclear localization of ERK^69^. As PKCε phosphorylates serine residue of the substrate protein, the phosphorylation of SPS sequence in ERK appear to contribute to PKCε-induced ERK activation. In this study, t-PKCε was detected in the nuclear fraction but not in the cytoplasm. Therefore, it is conceivable that such an interaction occurs on the nuclear side.

**Figure 8:**
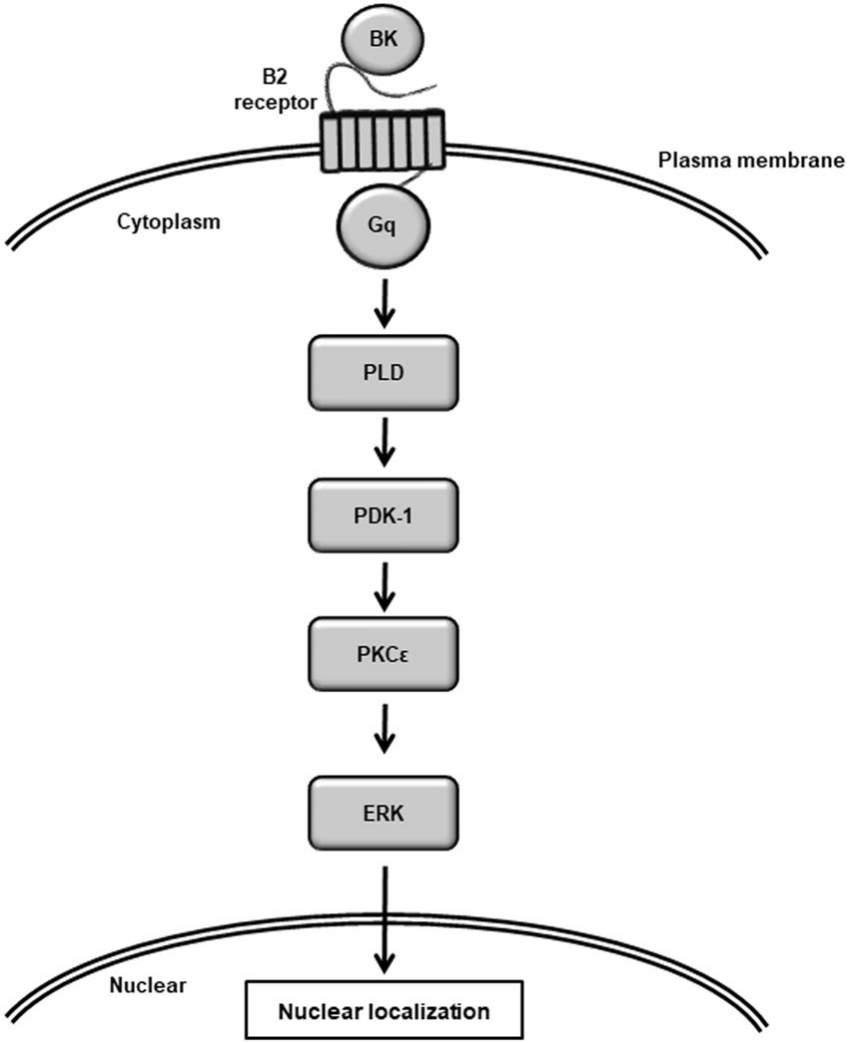
Schematic diagram of the intracellular signaling depicting bradykinin-induced COX-2 expression in dermal fibroblasts. Bradykinin (BK) induced the activation of PLD/PDK-1 axis via B2R and coupling G protein Gαq. The activated-PDK-1 induced the activation of PKCε, which leads to the nuclear accumulation of p-ERK1. Finally, the nuclear p-ERK1 contributes to COX-2 expression and prostaglandin E_2_ synthesis.

In conclusion, we demonstrated that bradykinin activates PLD/PDK-1 pathway via B2R and Gαq, which subsequently induces the activation of PKCε, ERK1 activation, and nuclear translocation are followed by the PKCε activation, and consequently induces COX-2 expression for prostaglandin E_2_ synthesis in dermal fibroblasts. A scheme consistent with the observations in bradykinin-induced dermal fibroblasts is provided in Fig. 8. Our observations will provide new insights into the signaling of inflammation evoked by bradykinin.

## Methods

### Materials

TRIzol, Alexa Fluor 594-conjugated F(ab′)2 fragments of goat anti-rabbit IgG (H + L), TO-PRO-3-iodide and ProLong Gold Antifade Reagent, Lipofectamine 2000, and Opti-MEM were purchased from Life Technologies Co. (Carlsbad, CA). CELLBANKER 1 plus medium, PrimeScript RT Master Mix, SYBR Premix Ex Taq II, Thermal Cycler Dice Real Time System II, and TP900 DiceRealTime v4.02B were obtained from TaKaRa Bio Inc. (Shiga, Japan). Rabbit monoclonal and polyclonal antibodies against mouse total PKCδ (t-PKCδ, EPR17075), human total PKCε (t-PKCε), human COX-1 (EPR5867), rabbit GAPDH (6C5), and human COX-2 and TGX221 were purchased from Abcam (Cambridge, UK). Rabbit monoclonal and polyclonal antibodies of anti-human phospho-ERK1/2 (p-ERK1/2, D13.14.4E), anti-rat total-ERK1/2 (t-ERK1/2, 137F5), anti-human phospho-PKCα (p-PKCα), anti-human phospho-PKCλ (p-PKCλ), anti-human total-PDK-1 (t-PDK-1), anti-human phospho-PDK-1 (p-PDK-1), and anti-human lamin A/C (4C11) and LY294002 were purchased from Cell Signaling Technology Japan, K.K. (Tokyo, Japan). Anti-human phospho-PKCε (p-PKCε) and anti-human phospho-PKCδ (p-PKCδ) rabbit polyclonal antibodies were purchased from Santa Cruz Biotechnology, Inc. (Dallas, TX). Mouse monoclonal anti-β-actin antibody (AC74), HOE140, R715, YM254890, H89, KT5823, BX795, FIPI, butanol, SB239063, FR180204, SP600125, U73122, and AS604250 were obtained from Sigma-Aldrich Inc. (St Louis, MO). Gallein was purchased from Tokyo Chemical Industry Co., Ltd. (Tokyo, Japan). Horseradish peroxidase-conjugated (HRP-conjugated) anti-rabbit and -mouse IgG antibodies, ECL Western Blotting Analysis System, ImageQuant LAS 4000 mini, and protein A/G plus Sepharose were purchased from GE Healthcare (Piscataway, NJ). iCycler, Mini-PROTEAN TGX gel, and polyvinylidene difluoride (PVDF) membranes were obtained from Bio-Rad (Hercules, CA). Complete mini EDTA-free protease inhibitor mixture and Block Ace were purchased from Roche (Mannheim, Germany). α-Modified Eagle Minimum essential medium (α-MEM) was purchased from Wako Pure Chemical Industries, Ltd. (Osaka, Japan). FR236924 was obtained from Tocris Bioscience (Bristol, UK). Calphostin C, Rottlerin, and an enzyme-linked immunosorbent assay (ELISA) kit for prostaglandin E_2_ were purchased from Cayman Chemical Co. (Ann Arbor, MI). Fura 2-AM was obtained from Dojindo Laboratories (Kumamoto, Japan). A freezing vessel BICELL was purchased from Nihon Freezer Co., Ltd. (Tokyo, Japan). StatMate IV was purchased from ATMS (Tokyo, Japan).

### Cell culture

This study was approved by Nihon University Animal Care and Use Committee (AP13B051). All experiments were performed in accordance with the relevant guidelines and regulations. Dog dorsal skin samples (n = 3, healthy 3-year-old beagle dogs, male) were collected after local anesthesia with 1% lidocaine and 10 μg/mL adrenaline. To minimize potential pain and infection, remifentanil hydrochloride (3 to 5 µg/kg/min; Janssen Pharmaceutical K.K, Tokyo, Japan) and cefazolin (22 mg/kg; Nichi-Iko Pharmaceutical Co., Ltd, Toyama, Japan) were administered intravenously before the time of awakening after anesthesia. Dermal fibroblasts were isolated by explant culture as reported previously^70^. Briefly, canine dermis from the dorsal skin was collected and cut into 3-mm^2^ sections. Each explant was placed into 90-mm Petri dish. The attached explants were maintained in a static-culture in an incubator at 5% CO_2_ and 37 °C using α-MEM supplemented with 10% fetal bovine serum (FBS). The medium was changed once a week and then, dermal fibroblasts were obtained as outgrowth cells. The cells were harvested using 0.25% trypsin-EDTA once they reached 90–95% confluence. The collected cells were suspended using CELLBANKER 1 plus medium at a density of 2 × 10^6^ cells/500 μL, and 500 μL of the cell suspension was placed into a sterilized serum tube. The tubes were then placed into a freezing vessel and cryopreserved at −80 °C. Before experiments, serum tubes were removed from the freezing vessel and immersed into a water bath at 37 °C. The thawed-out cell suspension was transferred into a centrifuge tube contained α-MEM containing 10% FBS and centrifuged at 300 *g* for 3 min. After removal of the supernatant, the pellet was suspended in α-MEM containing 10% FBS and transferred into a 75-cm^2^ culture flask. Static cultures were then maintained under the same conditions as before the cryopreservation. Cells were harvested using 0.25% trypsin-EDTA once they reached approximately 90% confluency. Then, the collected cells were seeded at a density of 1 × 10^6^ cells/75-cm^2^ culture flask. The fourth-passage canine dermal fibroblasts were used for all following experiments. The cells from different animals were used in different experiments. The cells were characterized by detecting the mRNA expression of chemotropic factors such as Netrin-1, Netrin-3, Ephrin-A3, Ephrin-A4, and Semaphorin-4D as reported previously^70^. The mRNA expression of chemotropic factors in dermal fibroblasts was less in comparison with mesenchymal stem cells, suggesting that the cells are dermal fibroblasts.

### RT-PCR

RT-PCR was performed as reported previously^55,71^. Total RNA was extracted from dermal fibroblasts using TRIzol reagent according to the manufacturer’s instructions. Total RNA concentration was measured spectrophotometrically by reading absorbance at 260/280 nm. First-strand cDNA synthesis was conducted using 500 ng of total RNA by using the PrimeScript RT Master Mix. PCR was performed using 2 µL of first-strand cDNA in 10 μL total reaction volume, with primers specific for PKCα, β, δ, ε, θ, η, ι/λ, ζ, and the TATA box binding protein (TBP), a house keeping protein as a control (see Supplementary Table S1), and Ex Taq. PCRs were conducted using iCycler. The thermal cycler was programmed for initial denaturation at 94 °C for 2 min, followed by 30 cycles of denaturation at 94 °C for 30 s, primer annealing at 55 °C for 30 s, and primer extension at 72 °C for 30 s. The PCR products were separated using 2% agarose gel electrophoresis, followed by ethidium bromide staining and visualization under UV light. mRNA expression levels in each sample were normalized to that of TBP.

### Real-time RT-PCR

Real-time PCR was performed as described previously^55,56,70–74^. Total RNA was extracted from dermal fibroblasts with TRIzol reagent. The first-strand cDNA synthesis was carried out with 500 ng of total RNA using PrimeScript RT Master Mix. Real-time RT-PCR was performed with 2 µL of the first-strand cDNA in 25 μL (total reaction volume) with SYBR Premix Ex Taq II and primers specific for COX-1, COX-2, and TBP (a house keeping protein used as a control) (see Supplementary Table S1). Real-time RT-PCR of no-template controls was performed with 2 µL of RNase- and DNA-free water. Additionally, real-time PCR of no-reverse transcription control was performed with 2 µL of each RNA sample. PCR was conducted using Thermal Cycler Dice Real Time System II using the following protocol: 1 cycle of denaturing at 95 °C for 30 s, 40 cycles of denaturing at 95 °C for 5 s and annealing/extension at 60 °C for 30 s. The results were analyzed by the second derivative maximum method and the comparative cycle threshold (ΔΔ Ct) method using real-time RT-PCR analysis software. The amplification of TBP from the same amount of cDNA was used as an endogenous control, while cDNA amplification from dermal fibroblasts at time 0 was used as a calibration standard.

### Western blotting

Western blotting was performed as reported previously^55,56,70–73^. The cells were lysed using a lysis buffer containing 20 mM HEPES, 1 mM PMSF, 10 mM sodium fluoride, and a complete mini EDTA-free protease inhibitor cocktail at pH 7.4. Protein concentrations were adjusted using the Bradford method^75^. Extracted proteins were boiled at 95 °C for 5 min in SDS buffer. Samples were loaded into separate lanes of 7.5% or 12% Mini-PROTEAN TGX gel and separated electrophoretically. Separated proteins were transferred to PVDF membranes, treated with Block Ace for 50 min at room temperature, and incubated with primary antibodies (COX-2 [1:1,000], COX-1 [1:100], p-PKCε [1:200], t-PKCε [1:200], t-PKCδ [1:200], p-PKCα [1:1,000], p-PKCλ [1:1,000], p-PKCp-PDK-1 [1:200], t-PDK-1 [1:200], p-ERK1/2 [1:1,000], t-ERK1/2 [1:1,000], lamin A/C [1:1,000], GAPDH [1:1,000] and β-actin [1:10,000]) for 120 min at room temperature. After washing, the membranes were incubated with an HRP-conjugated anti-rabbit or -mouse IgG antibody [1:10,000] for 90 min at room temperature. Immunoreactivity was detected using ECL Western Blotting Analysis System. Chemiluminescent signals were measured using ImageQuant LAS 4000 mini.

### Prostaglandin E_2_ assay

Dermal fibroblasts were seeded at a density of 3.0 × 10^5^ cells/ well in 6-well culture plates. These cells were treated with bradykinin after starvation for 24 h, and culture supernatants were collected. Prostaglandin E_2_ concentrations in the culture supernatant were measured using an ELISA kit according to the manufacturer’s instructions^55,70^.

### Immunoprecipitation

Total cell lysate (100 µg) was precleared with protein A/G plus Sepharose before incubation with specific antibodies, followed by addition of protein A/G plus Sepharose^55^. The total cell lysate was incubated with 5 µg anti-p- or t-PKCε antibody at 4 °C for 18 h. The precipitated proteins were dissolved and boiled at 95 °C for 5 min in SDS buffer before electrophoresis. Finally, the precipitated proteins were analyzed by western blotting.

### siRNA transfection

Dermal fibroblasts seeded at a density of 1 × 10^5^ cells/35-mm dish or 5 × 10^5^ cells/90-mm dish were transfected using Opti-MEM containing 5 μL/mL Lipofectamine 2000 and 50 nM PKCε, PKCδ, or scramble siRNA for 6 h^70^. Supplementary Table S2 shows sequences of siRNA. siRNA efficiency was determined by western blotting.

### Subcellular fractionation

Subcellular fractionation was performed as reported previously with slight modification^76,77^. Briefly, the cells were suspended in isotonic buffer containing 0.3 M sucrose, 10 mM HEPES, 2 mM EDTA, 0.25 mM EGTA, 1 mM PMSF, 10 mM sodium fluoride a complete mini EDTA-free protease inhibitor cocktail at pH 7.4. The cells were disrupted by repeated aspiration through a 27-gauge needle and centrifuged at 100,000 *g*, 4 °C for 60 min. The supernatant served as a crude cytosolic fraction sample. The pellet was suspended in isotonic buffer and centrifuged at 700 *g*, 4 °C for 5 min. The pellet containing crude nuclear fraction was washed three times and lysed with a lysis buffer containing 20 mM HEPES, 1 mM PMSF, 10 mM sodium fluoride, and a complete mini EDTA-free protease inhibitor cocktail at pH 7.4. Protein concentrations were adjusted using the Bradford method^75^. Extracted proteins were boiled at 95 °C for 5 min in SDS buffer. Finally, the proteins containing crude cytosolic or nuclear fraction were analyzed by western blotting.

### Immunocytochemistry

The protein localization was investigated by immunocytochemical analysis as reported previously^71–74^. Dermal fibroblasts were seeded at a density of 3 × 10^5^ cells/mL culture medium into a 35-mm glass bottom dish (Iwaki, Tokyo, Japan) treated with bradykinin. The cells were fixed with 4% paraformaldehyde (Nacalai Tesque Inc., Kyoto, Japan) for 15 min and processed for immunocytochemistry to examine the intra-cellular localization of p-ERK. The fixed cells were permeabilized by incubation with 0.2% Triton X-100 (Sigma-Aldrich Inc.) for 15 min at room temperature. Non-specific antibody reactions were blocked for 30 min with Block Ace (DS Pharma Biomedical, Osaka, Japan). The cells were then incubated for 90 min at room temperature with anti-p-ERK1/2 rabbit antibody [1:200]. After the cells were washed with PBS containing 0.2% polyoxyethylene (20) sorbitan monolaurate, they were incubated and visualized with Alexa Fluor 594-conjugated F(ab′)2 fragments of goat anti-rabbit IgG (H + L) [1:1,000] and TO-PRO-3-iodide [1:1,000] for 60 min in the dark at 25 °C. The cells were also incubated with only secondary antibodies as a control for nonspecific binding of the antibodies. These samples were washed thrice with PBS containing 0.2% polyoxyethylene (20) sorbitan monolaurate, dried, mounted with ProLong Gold Antifade Reagent, and visualized using a confocal laser scanning microscope (LSM-510; Carl Zeiss AG, Oberkochen, Germany). Co-localization analysis was performed using ZEN software (Carl Zeiss AG).

### Measurement of intracellular Ca^2+^ concentrations ([Ca^2+^]_i_)

The cells were pretreated with 2 μM Fura 2-AM in α-MEM supplemented with 10% FBS for 30 min at 37 °C. After the pretreatment, the Fura 2-loaded cells were trypsinized and suspended in α-MEM supplemented with 10% FBS. The Fura 2-loaded cells were put into quartz cuvettes containing Krebs-Ringer-HEPES solution containing 120 mM NaCl, 5 mM KCl, 1 mM MgCl_2_, 0.96 mM NaH_2_PO_4_, 0.2% glucose, 0.1% bovine serum albumin, 1 mM CaCl_2_, and 20 mM HEPES (pH 7.4) for the determination of [Ca^2+^]_i._ The fluorescence of Fura-2-loaded cells was measured with a CAF-110 spectrofluorometer (Nihon bunkou, Tokyo, Japan) with excitation at 340 nm and 380 nm and emission at 500 nm. The [Ca^2+^]_i_ was calculated from the ratio of the fluorescence intensities^27,33,34^.

### Statistical analysis

The data from these experiments are presented as the mean ± standard error of measurement. Statistical analysis was performed using StatMate IV. The data from the time course study were analyzed using two-way analysis of variance, and the data from other experiments were analyzed using one-way analysis of variance.

### Data availability

All data supporting the findings of this study are available from the corresponding author upon reasonable request.

## Electronic supplementary material

Supplementary information
