# Supplementary material for: Protein kinase Cε regulates nuclear translocation of extracellular signal-regulated kinase, which contributes to bradykinin-induced cyclooxygenase-2 expression
Source: Sci Rep. 2018 Jun 4;8:8535. doi: 10.1038/s41598-018-26473-7 (PMC5986758; doi:10.1038/s41598-018-26473-7)
Supplement: Supplementary file 1 — Supplementary information [file 41598_2018_26473_MOESM1_ESM.pdf]

Protein kinase Cε regulates nuclear translocation of extracellular signal-regulated kinase, which contributes to bradykinin-induced cyclooxygenase-2 expression

Rei Nakano, Taku Kitanaka, Shinichi Namba, Nanako Kitanaka & Hiroshi Sugiya

Figure 1f. first row original figure

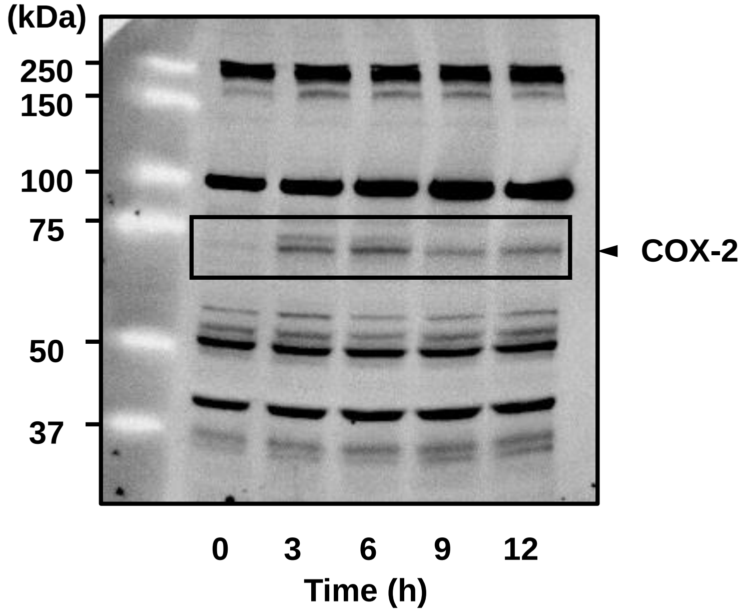

Figure 1f. second row original figure

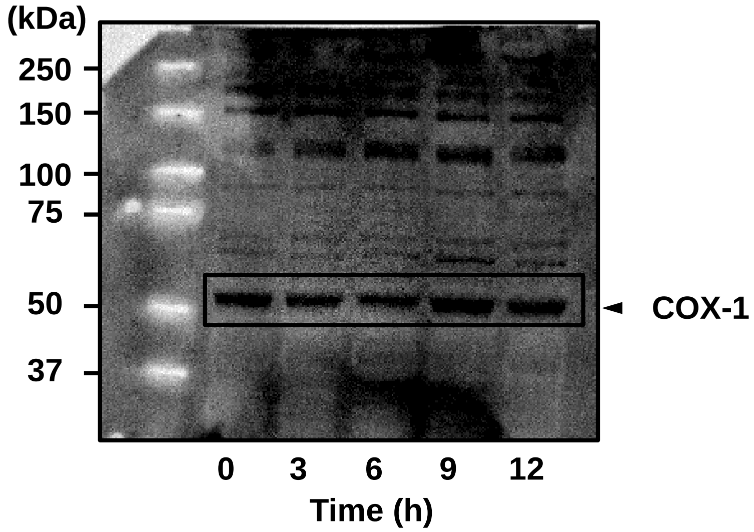

Figure 1f. third row original figure

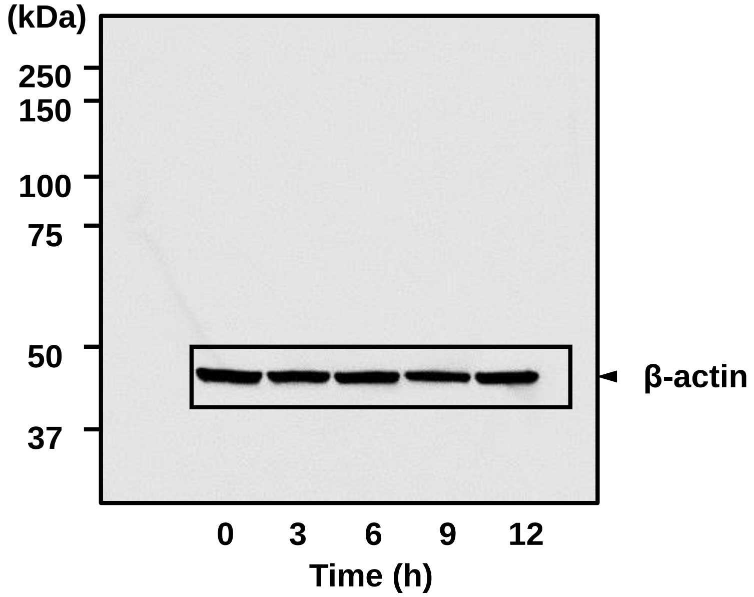

Supplementary Figure 1. Uncropped images for the blots shown in Figure 1f.

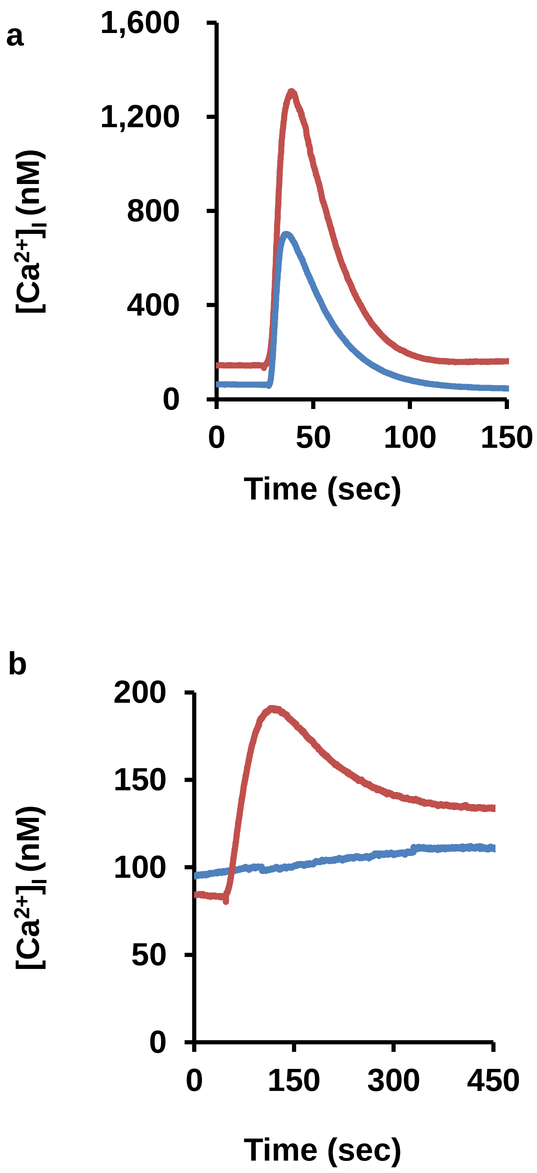

Supplementary Figure 2. Effects of bradykinin (BK), thapsogargin (TG) and phorbol 12-myristate 13-acetate (PMA) on $[Ca^{2+}]_i$ in Fura 2-loaded dermal fibroblasts. The cells were loaded with 2 μM Fura 2-AM for 30 min, and then stimulated with 1 μM BK, 5 μM TG or 100 nM PMA. **a.** In the cells stimulated with BK in the presence of extracellular Ca^{2+}, the basal $[Ca^{2+}]_i$ rapidly increased to a transient peak (1299.2 ± 352.9 nM) and then subsequently declined to a sustained level (red line). In the Ca^{2+}-free solution containing 0.5 mM EGTA, BK-induced transient peak $[Ca^{2+}]_i$ was reduced to 729.5 ± 88.5 nM and followed by a return to the basal level (blue line). **b.** In the cells stimulated with TG in the presence of extracellular Ca^{2+}, the basal $[Ca^{2+}]_i$ rapidly increased and then subsequently declined to a sustained level (red line) , whereas PMA had no effect on $[Ca^{2+}]$ (blue line). The results are representative of three independent experiments.

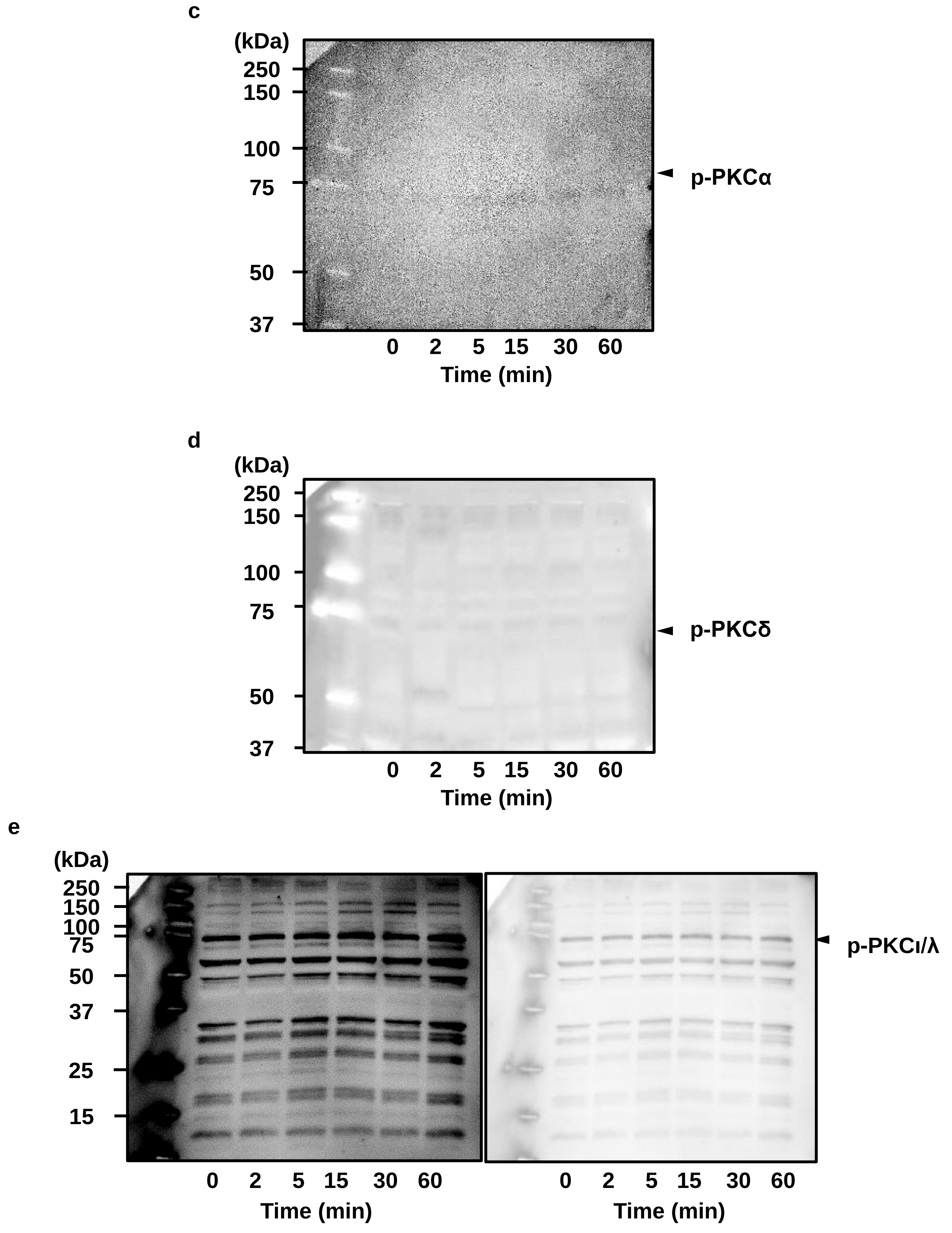

Supplementary Figure 2 (continue). The effect of bradykinin (BK) on the phosphorylation of PKCα, δ, ι/λ. The cells were treated with BK (1 μM) for the indicated time periods. BK had no effect on the phosphorylation levels of PKCα (c), δ (d) and ι/λ (e).

Figure 2f. original figure

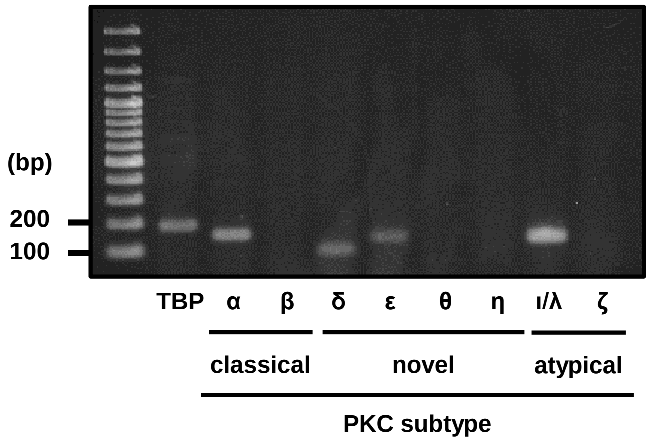

Figure 2g. original figure

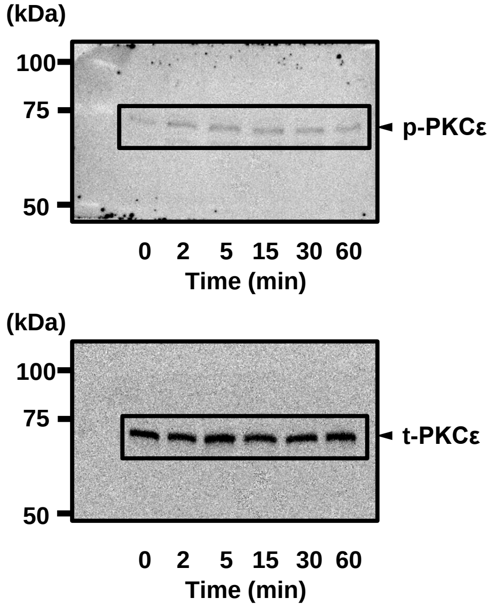

Figure 2i. original figure

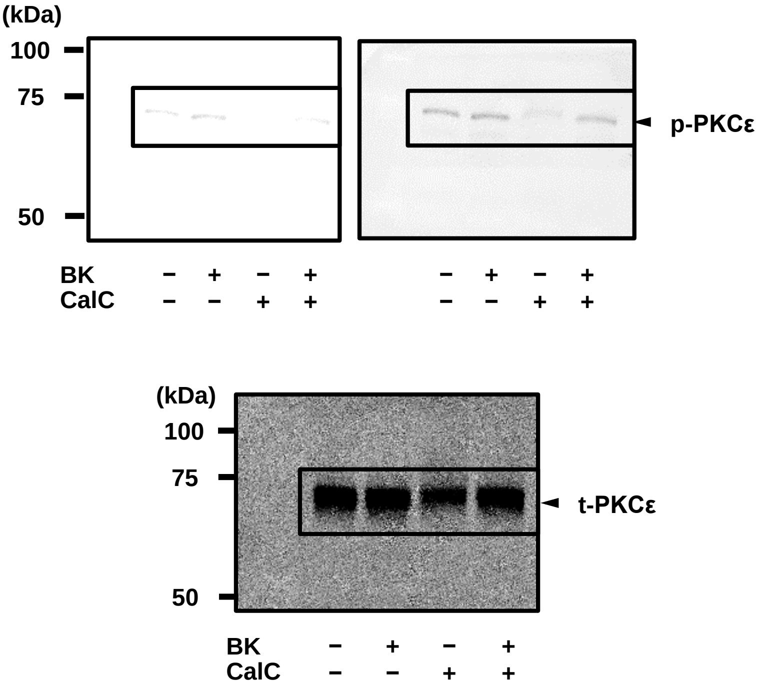

Figure 2k. original figure

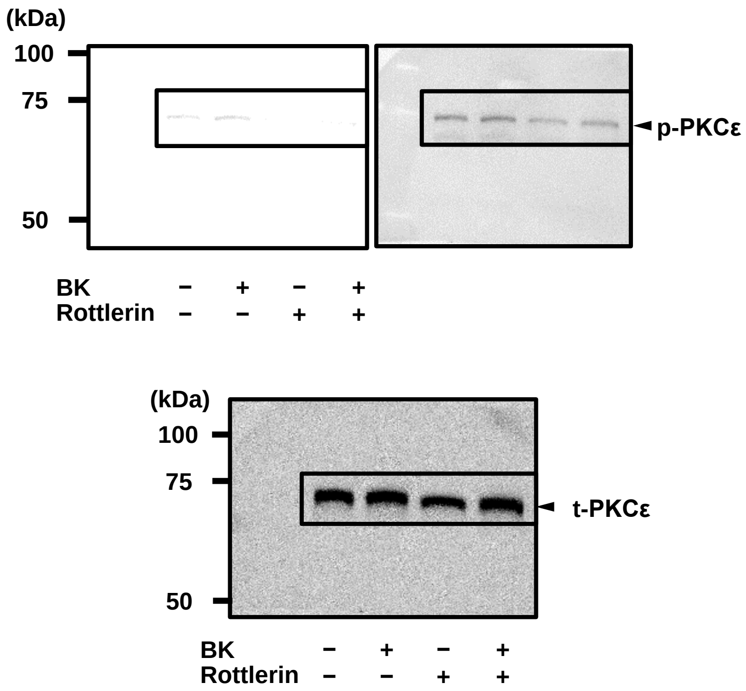

Figure 2n. original figure

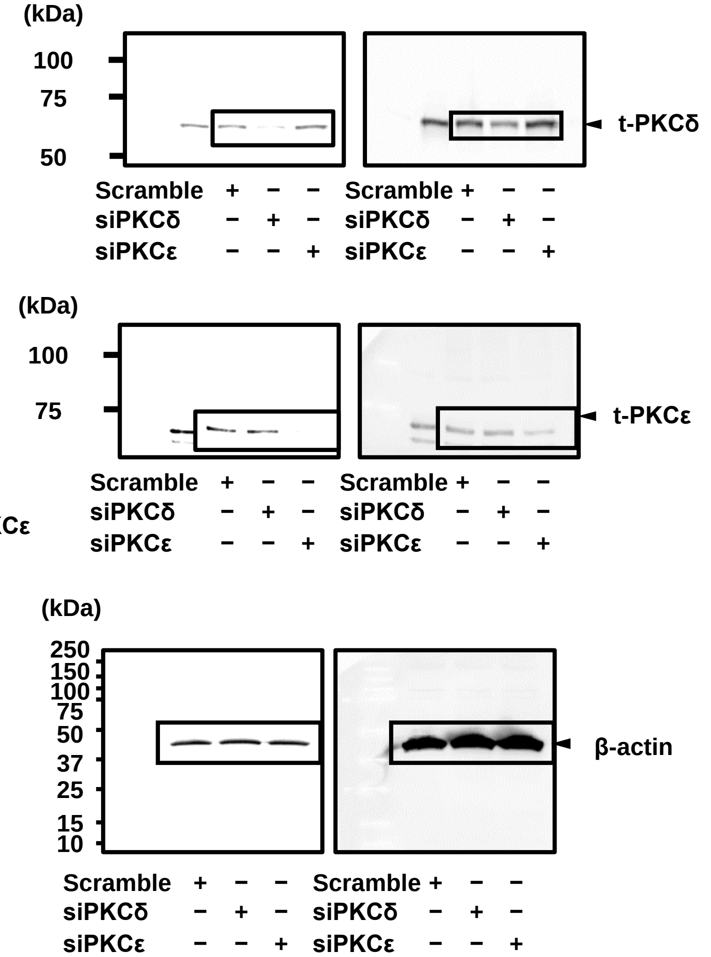

Supplementary Figure 2 (continue). Uncropped images for the blots shown in Figure 2.

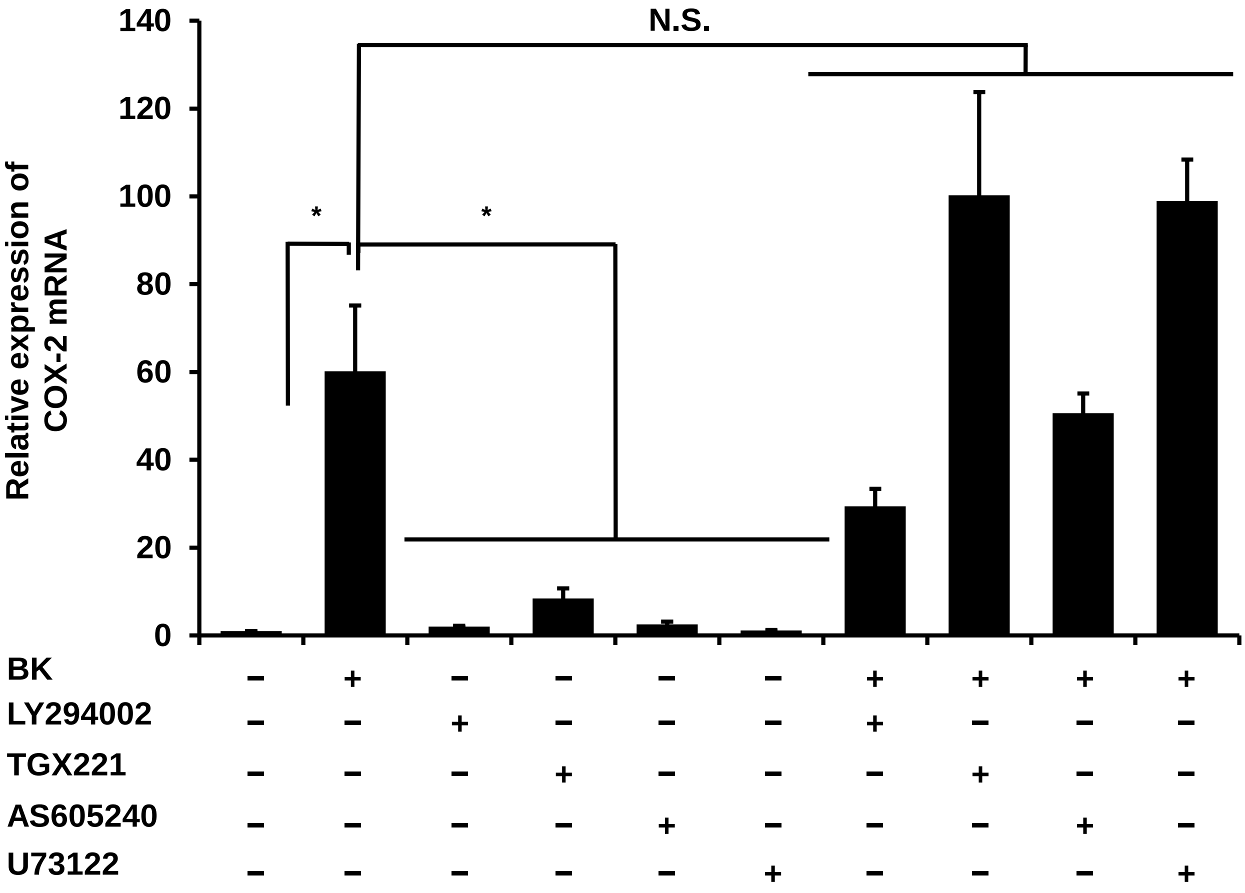

Supplementary Figure 3. Effect of inhibitors of pan-PI3K, PI3Kβ, PI3Kγ and PLC on the COX-2 mRNA expression induced by bradykinin (BK). After the pretreatment with the pan-PI3K inhibitor LY294002 (50 μM, 1 h), the PI3Kβ inhibitor TGX221 (20 μM, 30 min), the PI3Kγ inhibitor AS605240 (20 μM, 1 h) or the PLC inhibitor U73122 (8 μM, 1 h), the cells were incubated with 1 μM BK for 120 min. These inhibitors had no significant effect on the BK-induced COX-2 mRNA expression.

Figure 3b. original figure

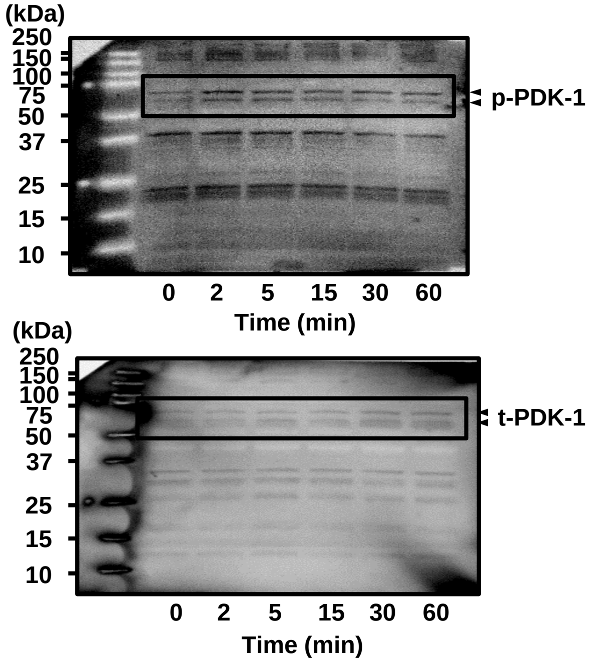

Figure 3i. original figure

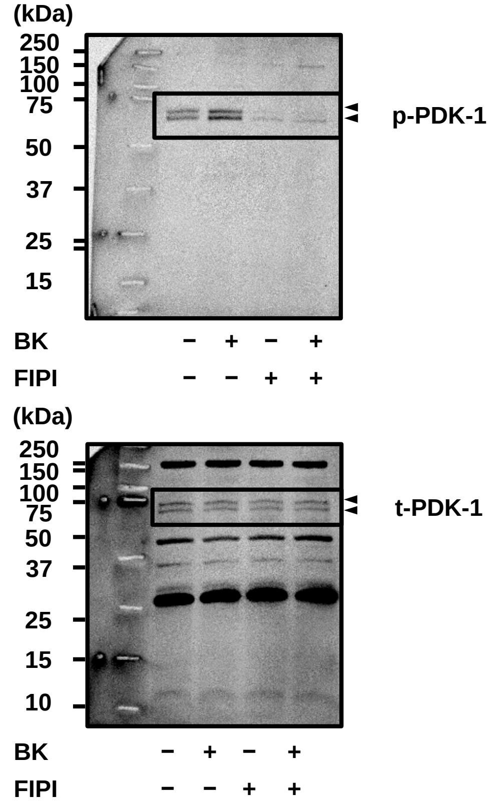

Figure 3d. original figure

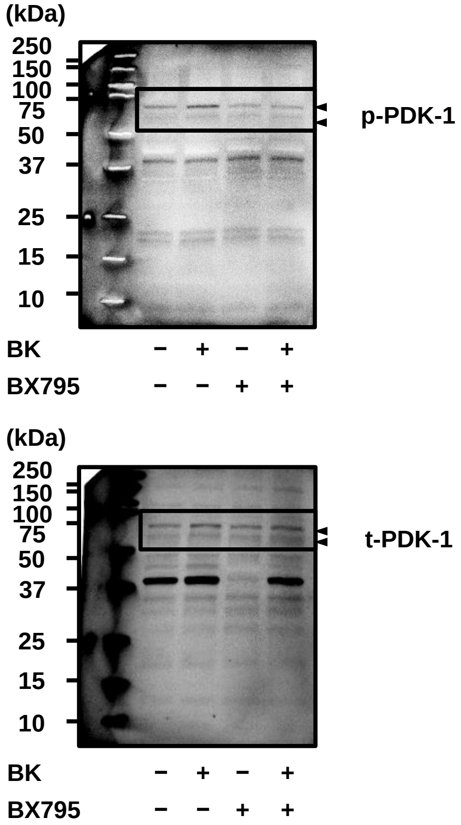

Figure 3k. original figure

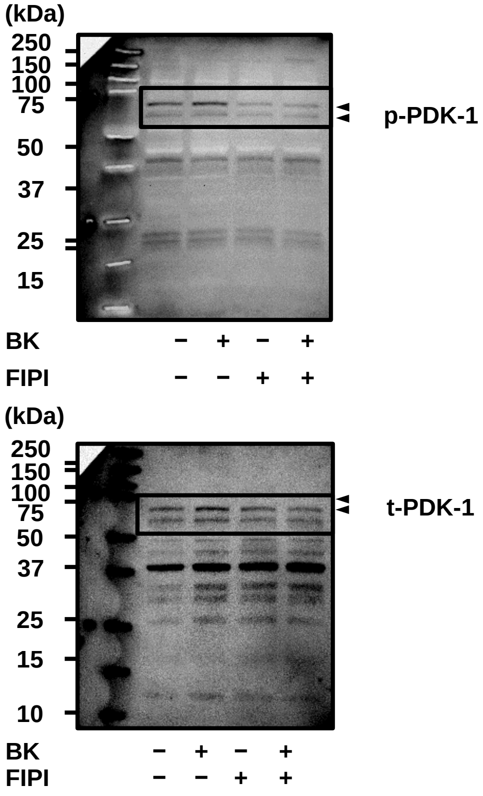

Figure 3f. original figure

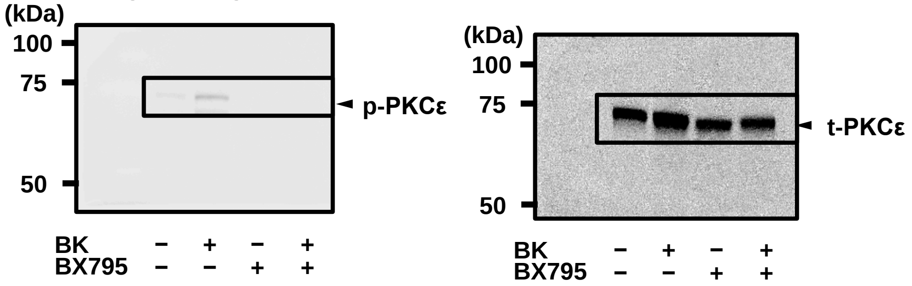

Supplementary Figure 3 (continue). Uncropped images for the blots shown in Figure 3.

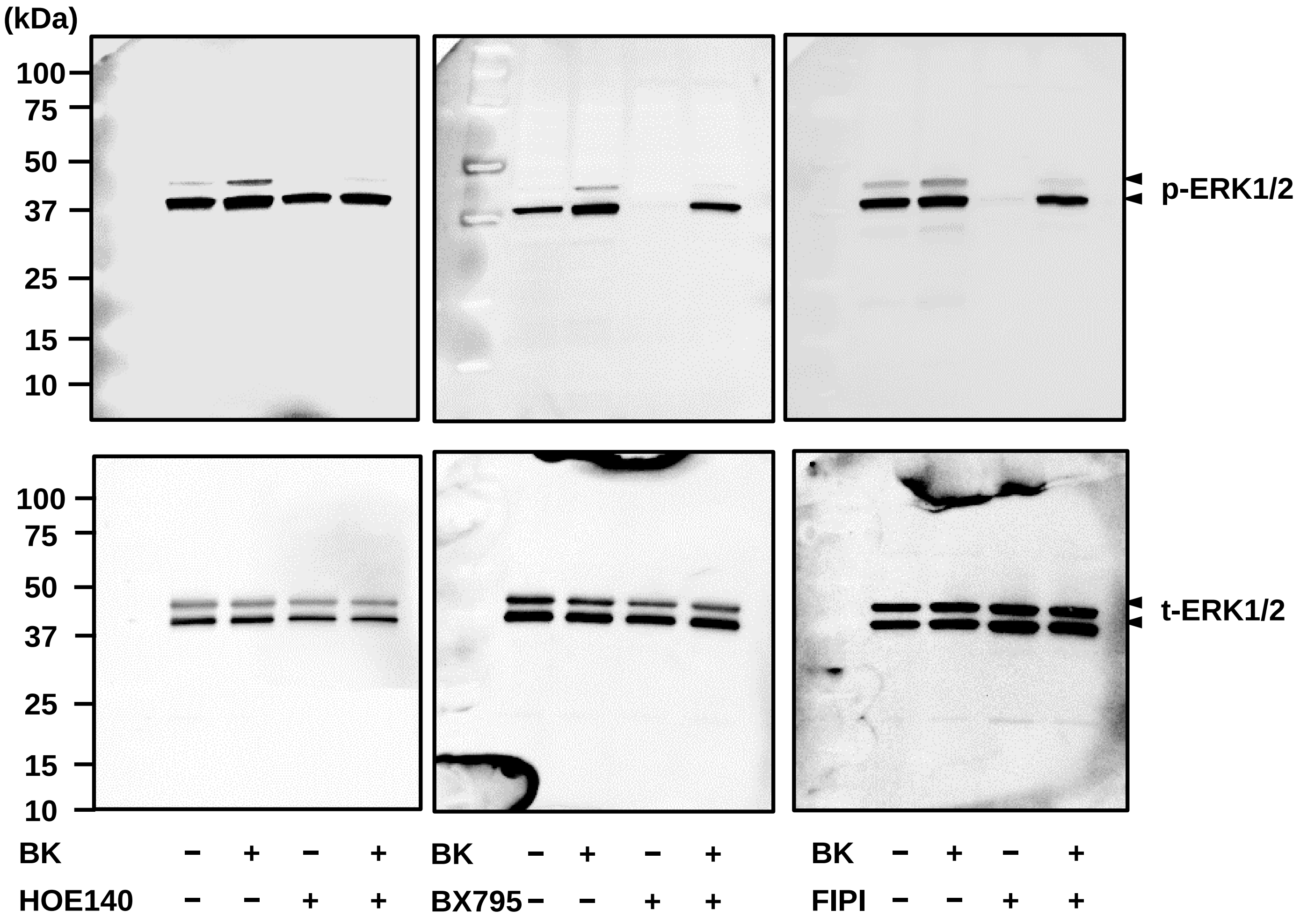

Supplementary Figure 4. Effect of B2R antagonist, PDK-1 inhibitor or PLD inhibitor on the ERK phosphorylation induced by bradykinin (BK). After the pretreatment with B2R antagonist HOE140 (5 μM, 1 min), PDK-1 inhibitor BX795 (30 μM, 1 h) or PLD inhibitor (50 μM, 1 h), the cells were incubated with 1 μM BK for 120 min. BK-induced ERK phosphorylation was clearly attenuated.

Figure 4b. original figure

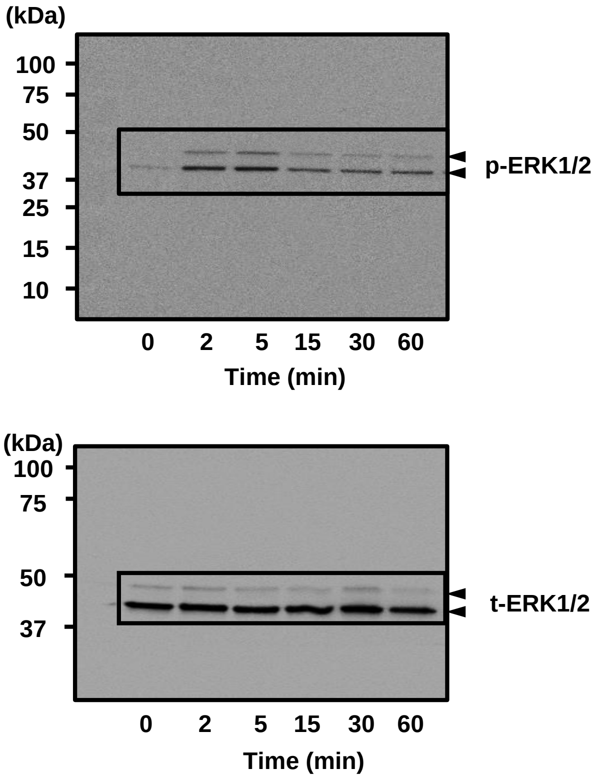

Figure 4f. original figure

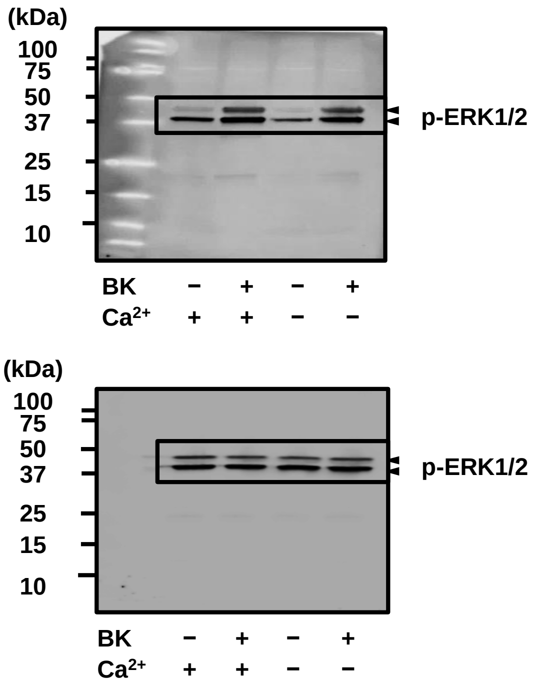

Figure 4d. original figure

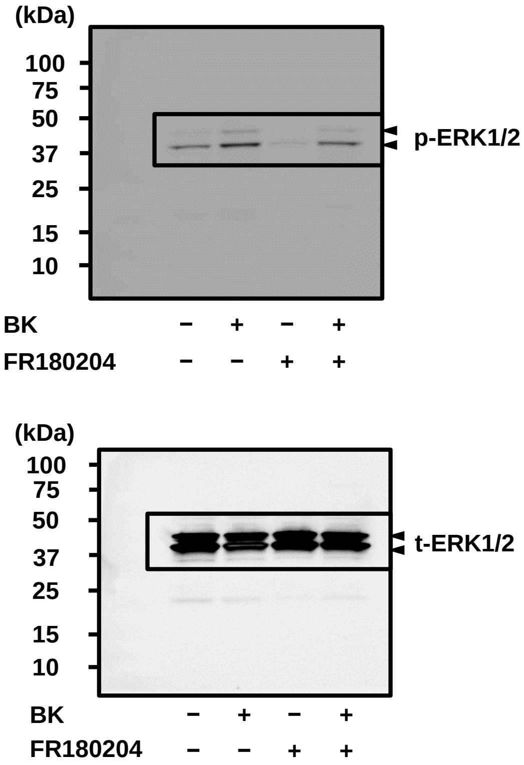

Figure 4h. original figure

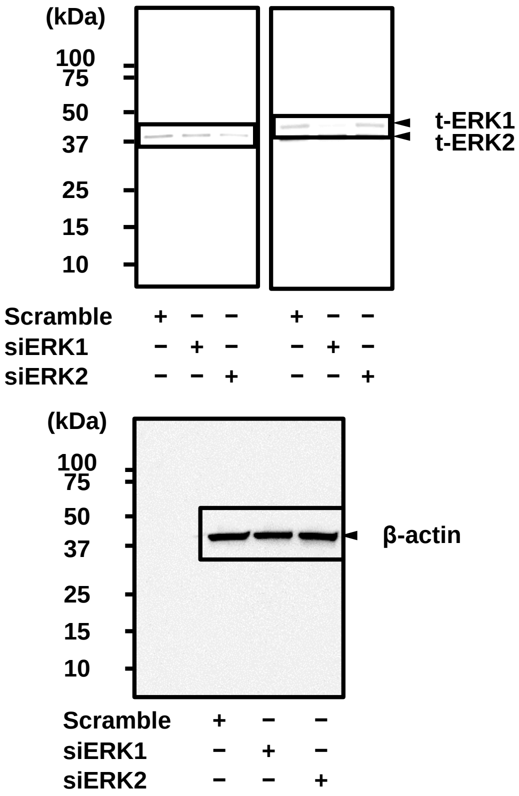

Supplementary Figure 4 (continue). Uncropped images for the blots shown in Figure 4.

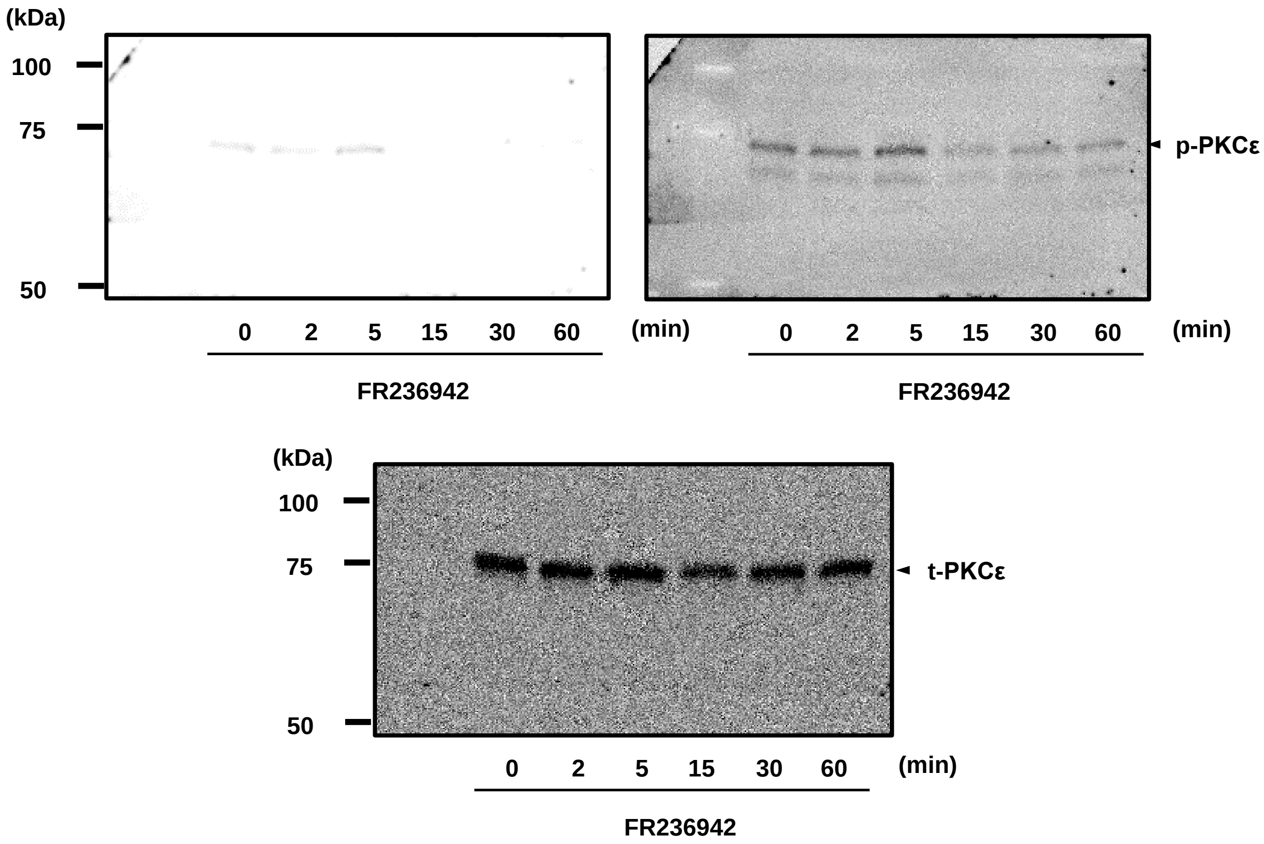

Supplementary Figure 5. Effect of a PKCε activator on the phosphorylation of PKCε. The cells were treated with the PKCε activator FR236942 for the indicated time periods. The PKCε activator time-dependently induced the expression of phosphorylated PKCε (p-PKCε; upper panel), whereas the expression of total PKCε (t-PKCε; lower panel) remained stable.

Figure 5a. original figure

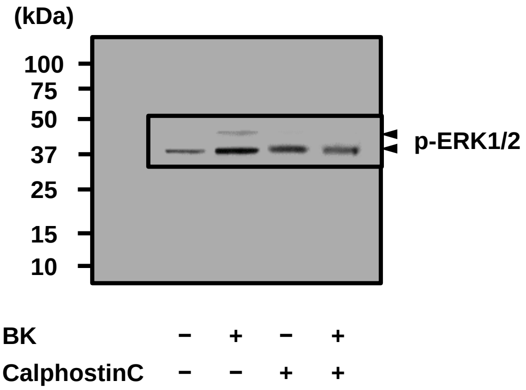

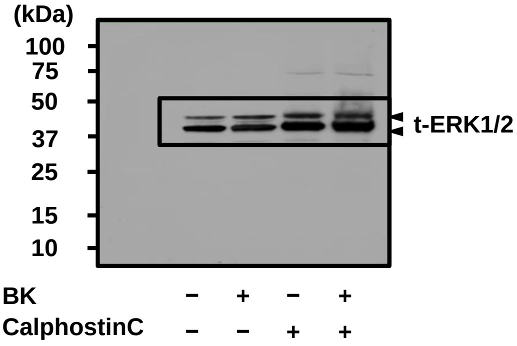

Figure 5c. original figure

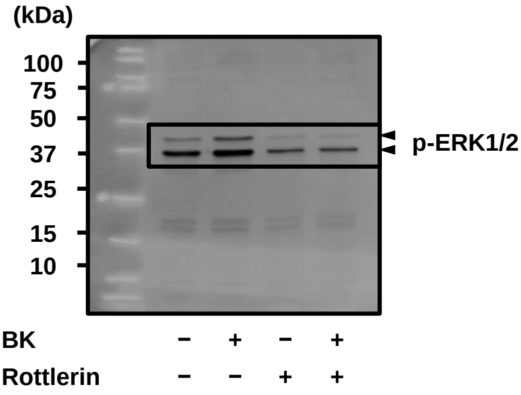

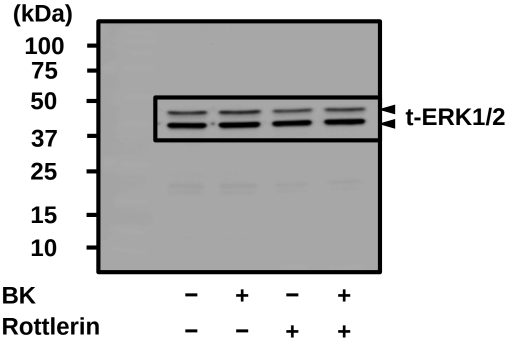

Supplementary Figure 5 (continue). Uncropped images for the blots shown in Figure 5.

Figure 5e. original figure

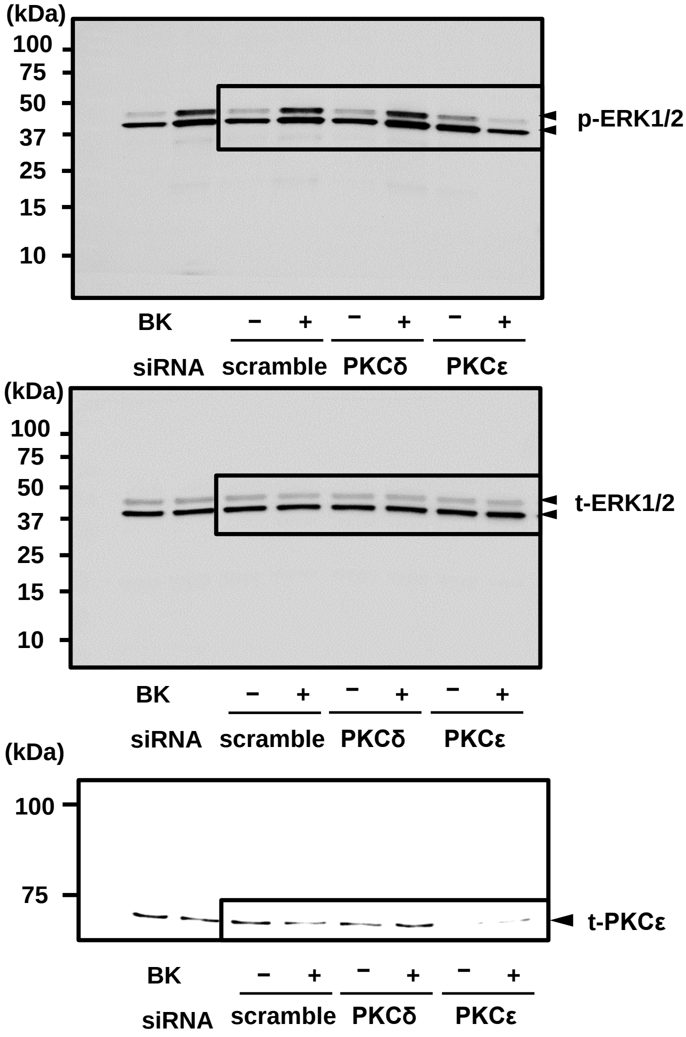

Figure 5i. original figure

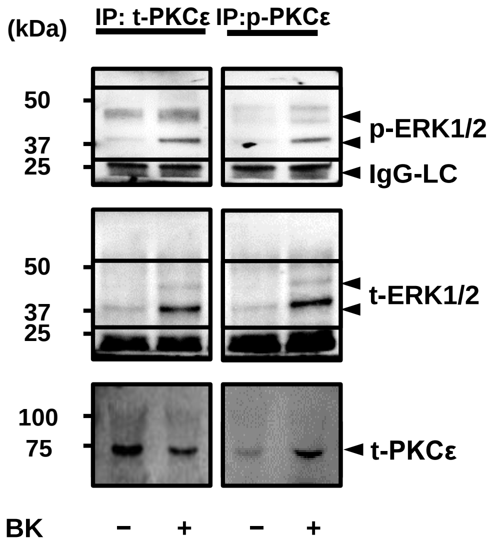

Figure 5g. original figure

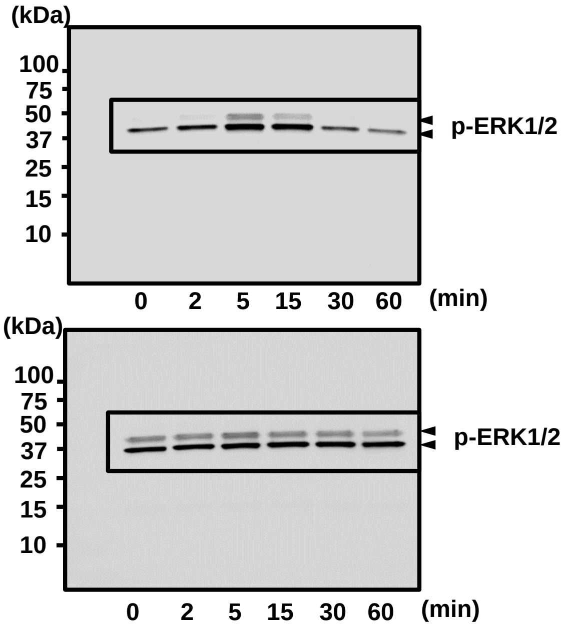

Supplementary Figure 5 (continue). Uncropped images for the blots shown in Figure 5.

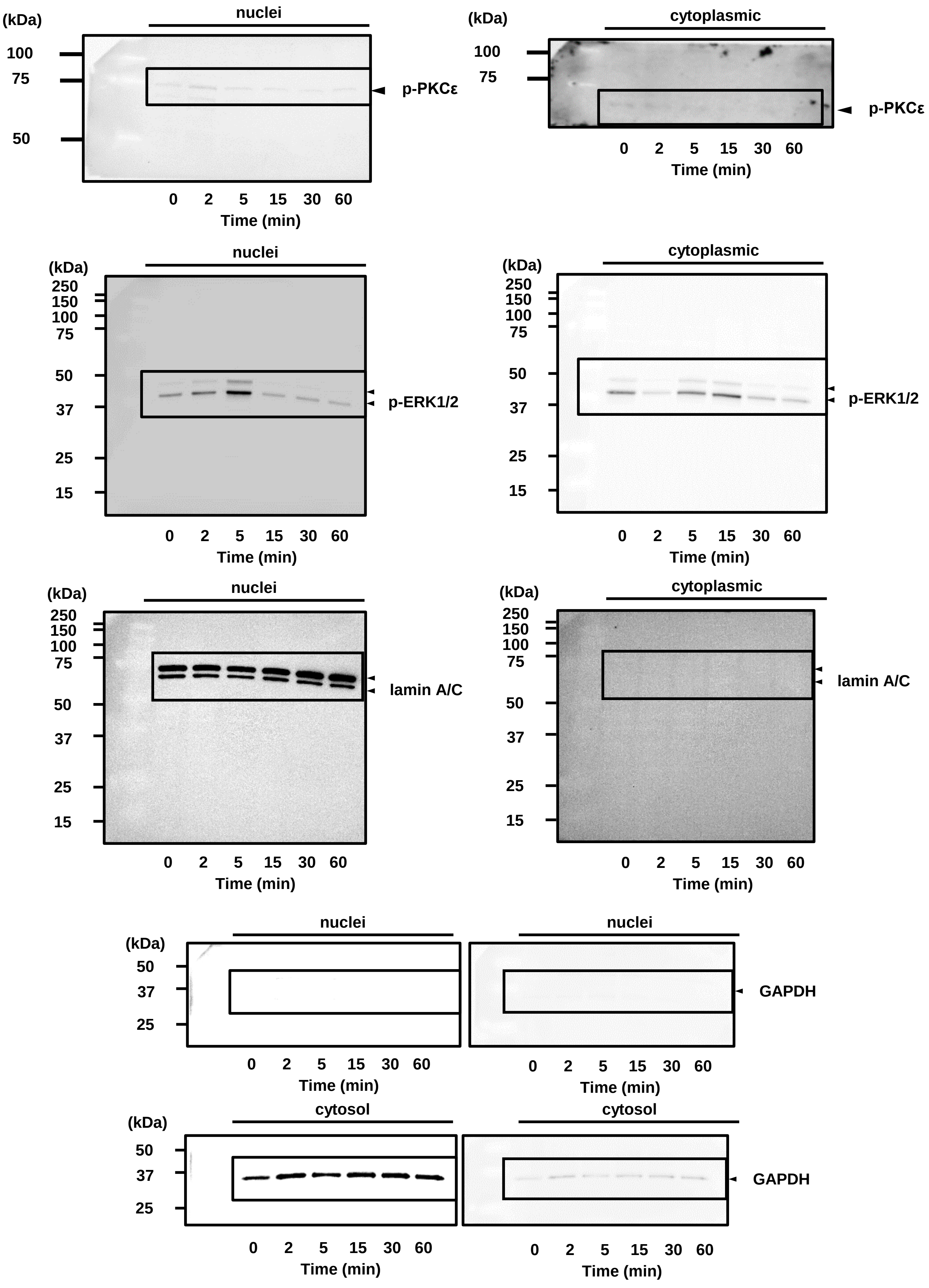

Supplementary Figure 6. Uncropped images for the blots shown in Figure 6.

Figure 6a. original figure

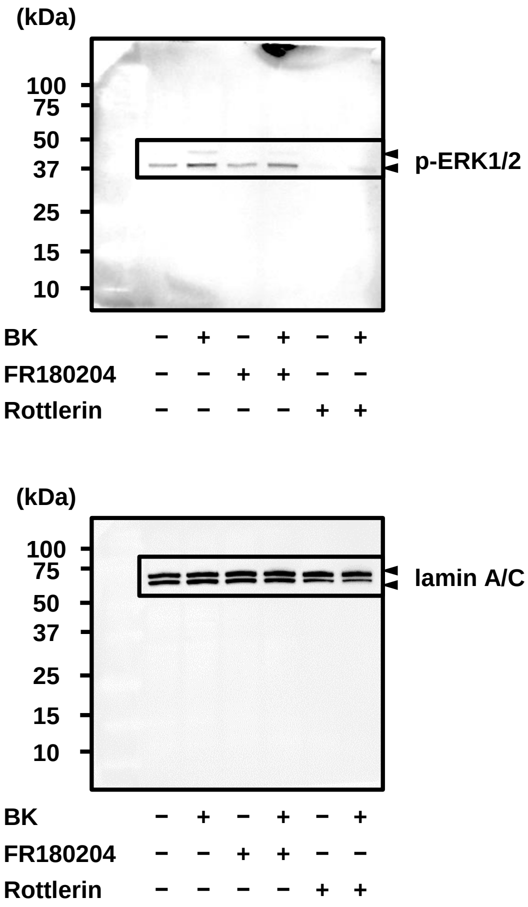

Figure 6e. original figure

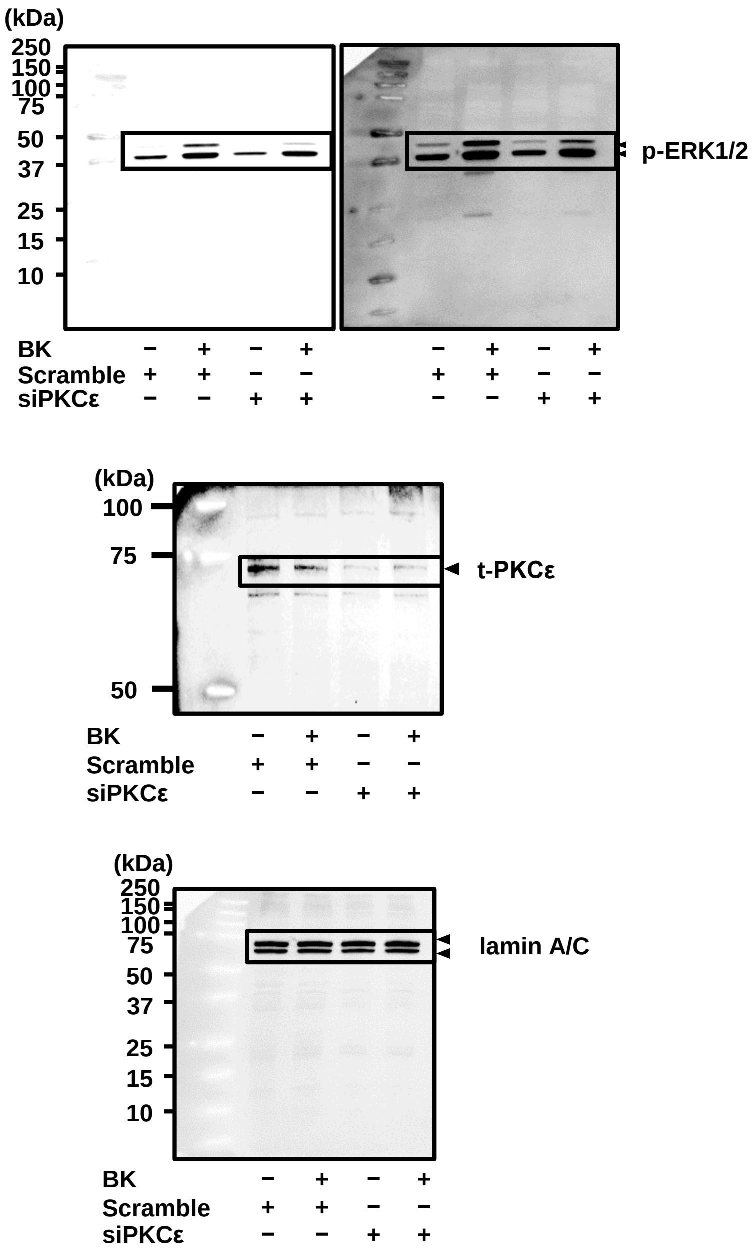

Supplementary Figure 7. Uncropped images for the blots shown in Figure 7.

Table S1 Primers used for RT-PCR or Real-time RT-PCR

Gene Name	Gene bank ID	Primer sequences
COX-2	NM_001003354.1	F: 5′-TGTGTCTCATTAACCTGCATGTACC-3′
		F: 5′-CAGTGATATTTGCACCTGTGTCCTC-3′
COX-1	NM_001003023.2	F: 5′-ACGTGGCTGTGGAAACCATC-3′
		R: 5′-GGCATCAATGTCTCCATACAGCTC-3′
PKCα	XM_022422857.1	F: 5′-CTTCTGCGGGACTCCAGATTAGA-3′
		R: 5′-AACAGCTCGTCTTCATCTTCACCA-3′
PKCβ	XM_022419944.1	F: 5′-TTTGGCAAGGTCATGCTCTCA-3′
		R: 5′-CATCGTACATTCCACATCGTCATC-3′
PKCδ	NM_001008716.1	F: 5′-TGCAGAAACTGCAATCCACAGA-3′
		R: 5′-CTCATGTACCACATCCAGGACAAAAG-3′
PKCε	XM_022424679.1	F: 5′-TGGCGTACCAGTGAAGTGATGAA-3′
		R: 5′-AATGAGATGATGCCACCATGAAGA-3′
PKCθ	XM_005617165.2	F: 5′-AGACTGAAACAGAGCGCCTCACC-3′
		R: 5′-TTACGCTTCCACAGATGCAAACA-3′
PKCη	XM_547844.6	F: 5′-GCGAACGTGGCACCTAACTG-3′
		R: 5′-AAACGAGCTTCGAGGTTGGAGA-3′
PKCι/λ	XM_535855.6	F: 5′-AAGCCAAGCGTTTCAACAGG-3′
		R: 5′-TGACGAGTTTGTGGCACTTC-3′
PKCζ	XM_843999.5	F: 5′-ACGGCCACATCAAGTTAACG-3′
		R: 5′-ATTTGGGGTTCCGCAGAAAG-3′
TBP	XM_863452	F: 5'-ACTGTTGGTGGGTCAGCACAAG-3'
		R: 5'-ATGGTGTGTACGGGAGCCAAG-3'

Table S2 Sequences for siRNA transfection

Gene Name	Gene bank ID	siRNA sequences
PKCδ	NM_001008716.1	F: 5'-CGUUCUUCAAGACCAUUAA-3'
		R: 5'-UUAAUGGUCUUGAAGAACG-3'
PKCε	XM_022424679.1	F: 5'-CCAUCAAGCAGCACCCAUU-3'
		R: 5'-AAUGGGUGCUGCUUGAUGG-3'
